# Supplementary material for: Syn-tasiR-VIGS: virus-based targeted RNAi in plants by synthetic trans-acting small interfering RNAs derived from minimal precursors
Source: Nucleic Acids Res. 2025 Mar 19;53(5):gkaf183. doi: 10.1093/nar/gkaf183 (PMC11920798; doi:10.1093/nar/gkaf183)
Supplement: gkaf183_Supplemental_Files [file gkaf183_supplemental_files.zip › Supplemental_all.pdf]

SUPPLEMENTARY DATA

Supplementary Data are available at NAR Online.

Data S1. sRNA reads from art-sRNA-expressing tissues.

Figure S1. Direct syn-tasiRNA cloning in *B/c* (*Bsa*I/*ccd*B)-based vectors including a *ccd*B cassette flanked by two *Bsa*I sites.

Figure S2. Direct syn-tasiRNA cloning downstream the AtmiR173a or NbmiR482a target sites (TSs) in *B/c* (*Bsa*I/*ccd*B)-based vectors including a *ccd*B cassette flanked by two *Bsa*I sites.

Figure S3. Functional analysis of syn-tasiRNAs against *N. benthamiana SULPHUR* (*NbSu*) expressed from modified minimal precursors including different miRNA target sites (TS).

Figure S4. Analysis of NbmiR482a and NbmiR6019a presence in *Nicotiana benthamiana* tissues.

Figure S5. Mapping of 19-24-nucleotide small RNA reads to min_{482}*-Su* precursors.

Figure S6. Functional analysis of potato virus X (PVX) constructs expressing syn-tasiR-NbSu from minimal precursors including endogenous or heterologous 22-nt miRNA target sites (TSs).

Figure S6. Functional analysis of potato virus X (PVX) constructs expressing syn-tasiR-Su from minimal precursors including endogenous or heterologous 22-nt miRNA target sites (TSs).

Figure S7. Functional analysis of tobacco rattle virus (TRV) constructs expressing syn-tasiR-Su from minimal syn-tasiRNA precursors in *N. benthamiana*.

Figure S8. *B/c*-based vectors for direct cloning of syn-tasiRNAs downstream the AtmiR173a and NbmiR482a target sites (TSs), or the TS of choice.

Table S1. Name, sequence and use of DNA oligonucleotides used in this study.

Table S2. Main features of DNA constructs functionally analyzed *in vivo* in this study.

Table S3: Phenotypic penetrance of syn-tasiRNAs expressed in *A. thaliana* Col-0 T1 transgenic plants for silencing *FT*.

Table S4: Phenotypic penetrance of syn-tasiRNAs expressed in *A. thaliana* Col-0 T1 transgenic plants for silencing *CH42*.

Table S5: Phenotypic penetrance of syn-tasiRNAs expressed in Arabidopsis Col-0 T1 transgenic plants for silencing *FT* and *TRY*.

Text S1. Protocol to design and clone syn-tasiRNAs downstream the 3'D1[+] position in *Bsa*I/*ccd*B-based ('B/c') vectors *pENTR-B/c, pMDC32-B/c, pENTR-AtmiR173aTS-B/c, pMDC32B-AtmiR173aTS-B/c*, *pENTR-NbmiR482aTS-B/c* and *pMDC32B-NbmiR482aTS-B/c*.

Text S2. Protocol to generate PVX-based syn-tasiRNA constructs.

Text S3. DNA sequence in FASTA format of all precursors used to express art-sRNAs in plants.

Text S4. DNA sequence of *Bsa*I-*ccd*B-based (B/c) vectors used for direct cloning of syn-tasiRNAs.

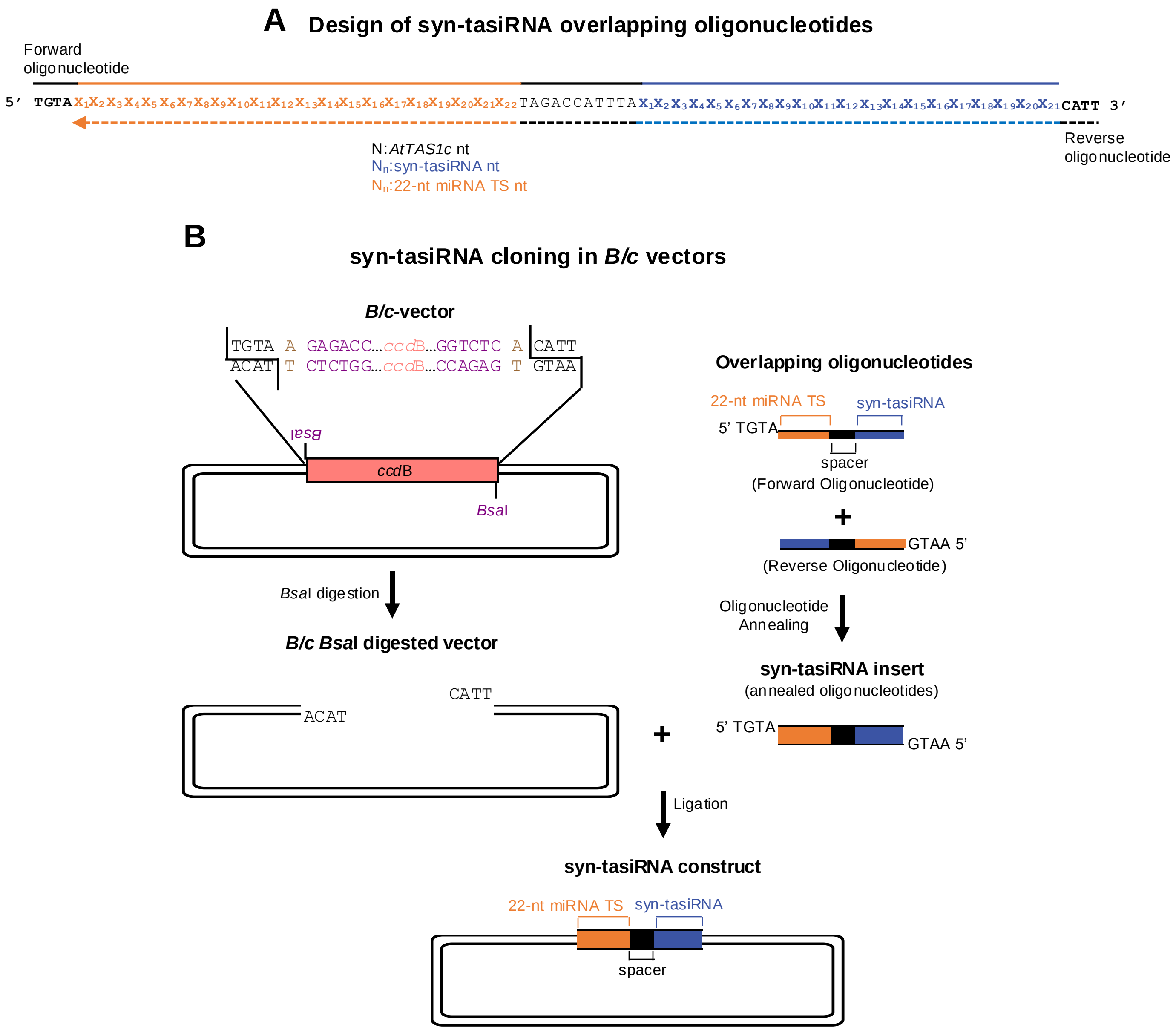

Figure S1. Direct syn-tasiRNA cloning in *B/c* (*Bsa*I/*ccd*B)-based vectors including a *ccd*B cassette flanked by two *Bsa*I sites. (**A**) Design of two overlapping oligonucleotides for syn-tasiRNA cloning. Sequence covered by the forward and reverse oligonucleotides are represented with continuous or dotted lines, respectively. Nucleotides of the 22-nt miRNA target site, the *AtTAS1c*-derived spacer and the syn-tasiRNA sequences are in orange, black and blue, respectively. Oligonucleotide 5' overhangs are in black and bold. (**B**) Diagram of the steps for syn-tasiRNA cloning in *B/c* vectors. The syn-tasiRNA insert, including the 22-nt miRNA target site sequence of interest followed by a 11-nt *AtTAS1c*-derived spacer, obtained after annealing the two overlapping oligonucleotides has 5' TGTA and 5'-AATG overhangs and is directly inserted into the *Bsa*I-linearized *B/c* vector. Nucleotides of the *Bsa*I sites and arbitrary nucleotides used as spacers between the *Bsa*I recognition site and the *AtTAS1c* sequence are in purple and light brown, respectively. Other details are as in A.

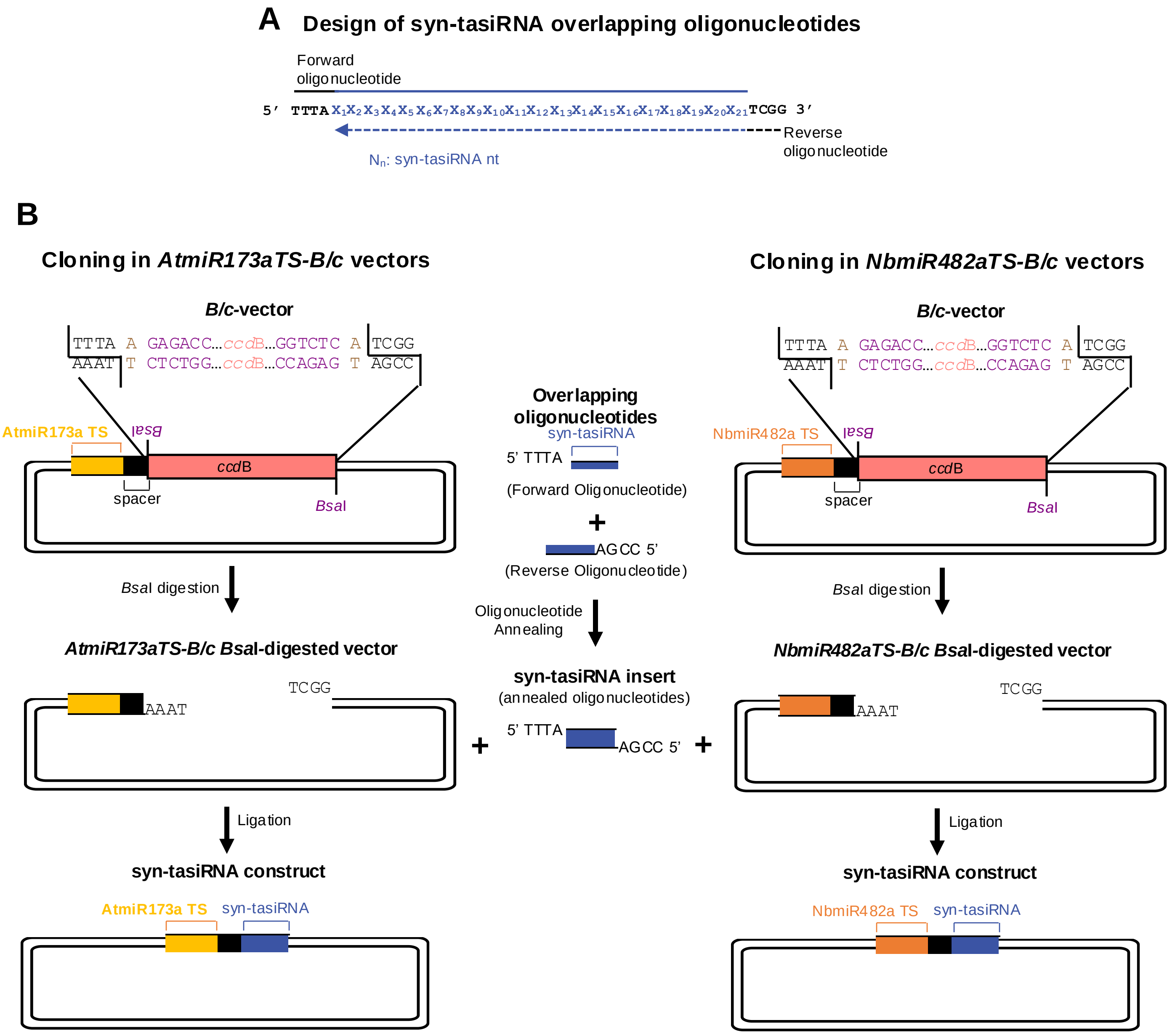

Figure S2. Direct syn-tasiRNA cloning downstream the AtmiR173a or NbmiR482a target sites (TSs) in *B/c* (<u>*Bsa*</u>I/<u>*ccd*</u>B)-based vectors including a *ccd*B cassette flanked by two *Bsa*I sites. (**A**) Design of two overlapping oligonucleotides for syn-tasiRNA cloning. Sequence covered by the forward and reverse oligonucleotides are represented with continuous or dotted lines, respectively. Nucleotides of the syn-tasiRNA sequence are in blue, and oligonucleotide 5' overhangs are in black and bold. (**B**) Diagram of the steps for syn-tasiRNA cloning in *AtmiR173aTS-B/c* or *NbmiR482aTS-B/c* vectors. The syn-tasiRNA insert obtained after annealing the two overlapping oligonucleotides has 5' TTTA and 5'-CCGA overhangs and is directly inserted into the *Bsa*I-linearized *AtmiR173aTS-B/c*- or *NbmiR482aTS-B/c*-based vectors. Nucleotides of the *Bsa*I sites and arbitrary nucleotides used as spacers between the *Bsa*I recognition site and the *AtTAS1c* sequence are in purple and light brown, respectively. Nucleotides of the AtmiR173a and NbmiR482a target sites are in yellow and orange, respectively. The *AtTAS1c*-derived spacer and the syn-tasiRNA sequences are in black and blue, respectively.

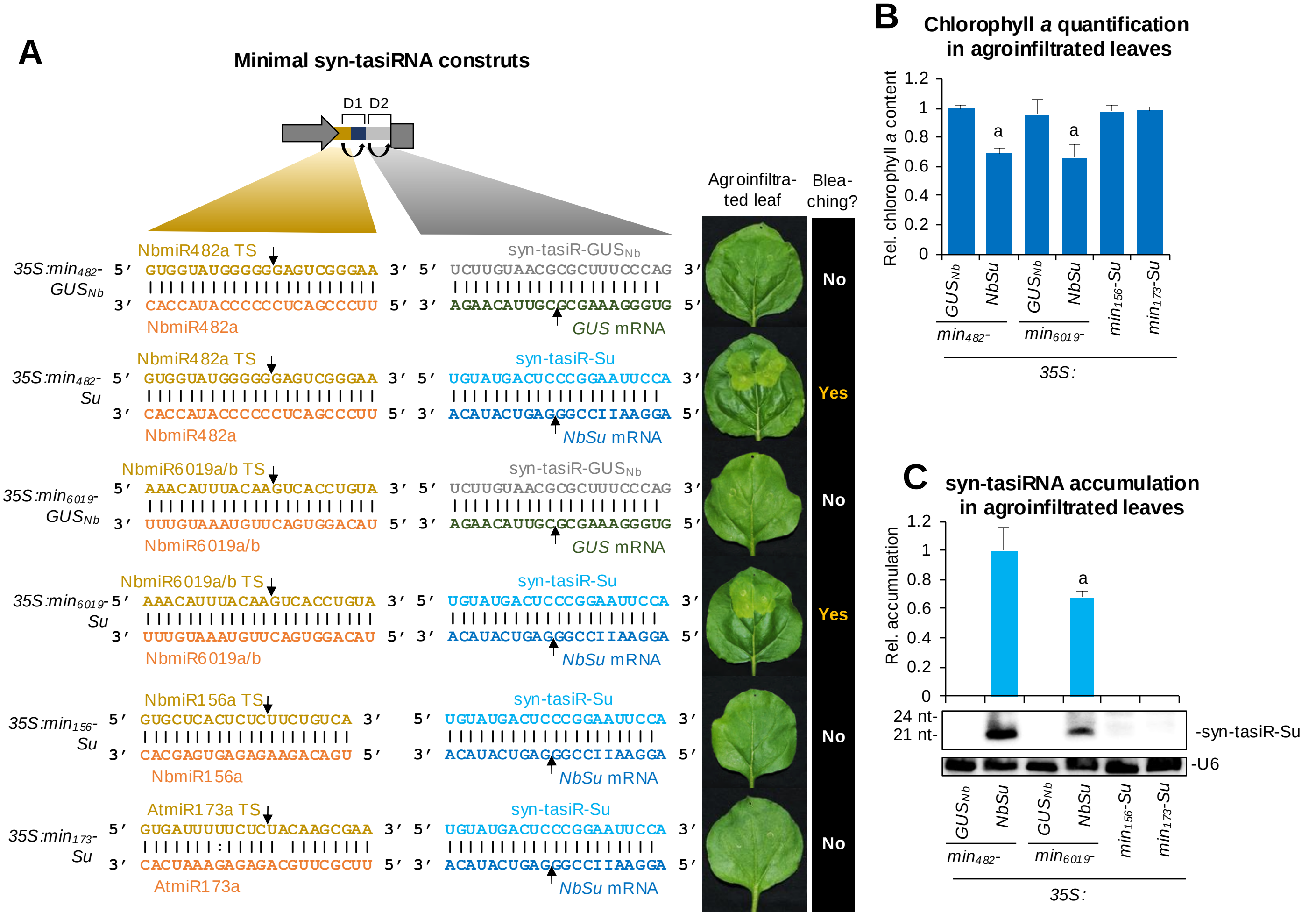

Figure S3. Functional analysis of syn-tasiRNAs against *N. benthamiana SULPHUR* (*NbSu*) expressed from modified minimal precursors including different miRNA target sites (TS). (**A**) Organization of minimal syn-tasiRNA constructs. Left, Minimal syn-tasiRNA constructs including 22-nt miRNA target sites from *N. benthamiana* (NbmiR482a TS and NbmiR6019 TS) or *A. thaliana* (AtmiR173a), or the 21-nt TS from *N. benthamiana* miR156a (NbmiR156a TS). Nucleotides corresponding to miRNA TSs and miRNAs are shown in dark yellow and orange, respectively. Nucleotides corresponding to syn-tasiR-NbSu and target *NbSu* mRNA are shown in light and dark blue, respectively. Nucleotides corresponding to syn-tasiR-GUS_{Nb} and *GUS* mRNA are shown in grey and dark green, respectively. Arrows indicate the predicted cleavage sites for miRNAs and syn-tasiRNAs. Right: photographs at 7 days post agroinfiltration (dpa) of leaves agroinfiltrated with each of construct. The presence or absence of bleaching on the agroinfiltrated patches is labelled as “Yes” or “No”, respectively. (**B**) Relative content of chlorophyll *a* in agroinfiltrated patches (*35S:min*$_{482}$-GUS_{Nb} = 1.0). Bars with letter “a” are significantly different from the control sample (P < 0.05 in pairwise Student’s t -test comparisons). (**C**) Target *NbSu* mRNA accumulation in agroinfiltrated leaves at 2 dpa [mean relative level (n = 3) + standard error] after normalization to *PROTEIN PHOSPHATASE 2A* (*NbPP2A*), as determined by quantitative RT-qPCR (*35S:min*$_{482}$-GUS_{Nb} = 1). Other details are as in B.

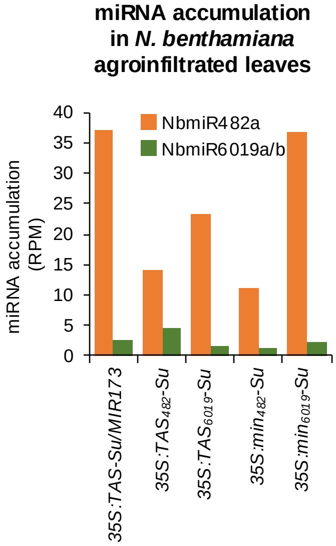

Figure S4. Analysis of NbmiR482a and NbmiR6019a/b presence in *Nicotiana benthamiana* tissues. Left, bar graph showing the accumulation (reads per million, RPM) of NmiR482a and NbmiR6019a/b revealed by high-throughput sequencing of small RNA libraries prepared from leaves agroinfiltrated with different constructs.

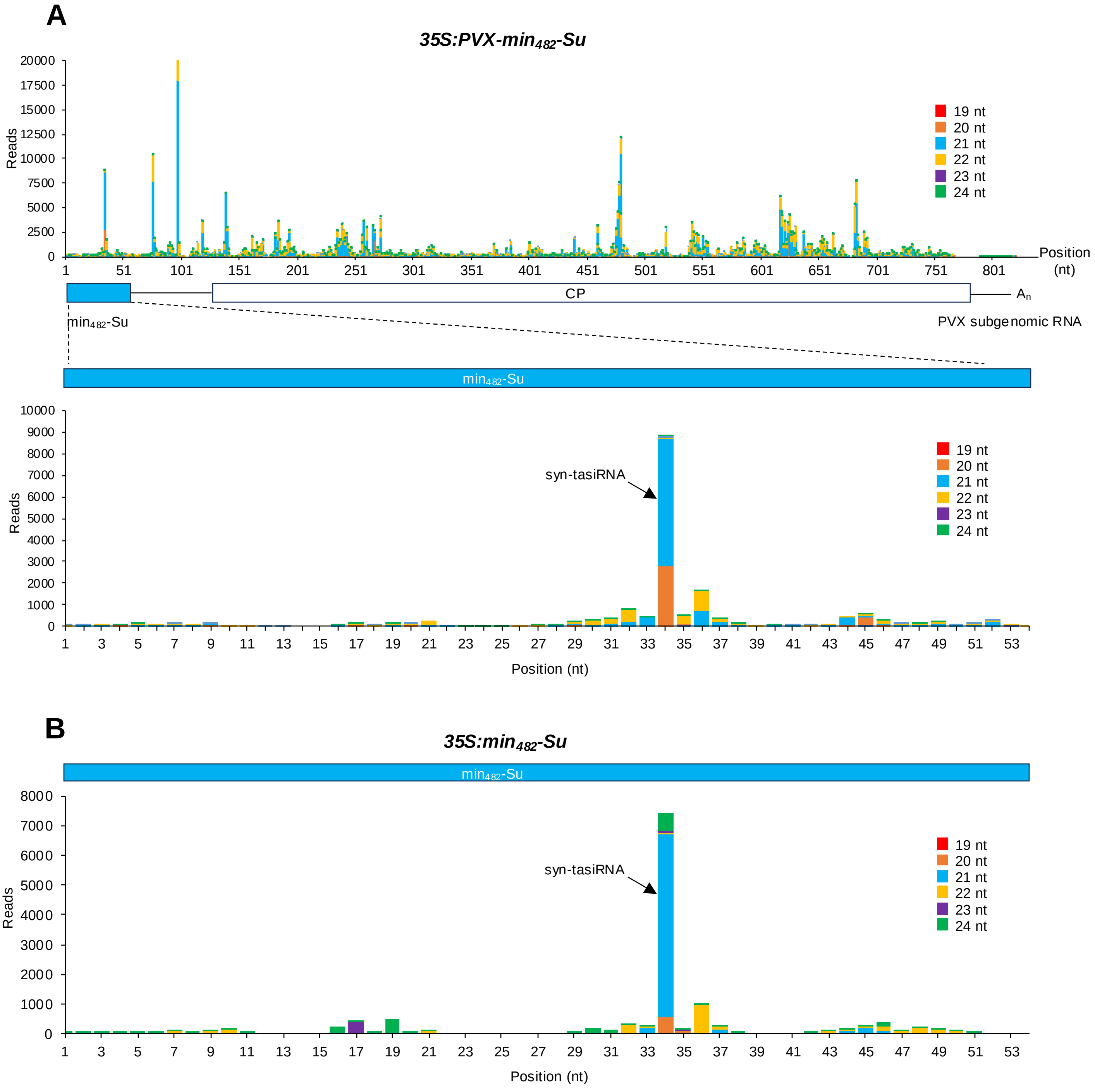

Figure S5. Mapping of 19-24-nucleotide small RNA reads to *min$_{482}$-Su* precursors. **A)** Top, mapping of reads to the whole subgenomic RNA sequence including PVX coat protein (CP). Bottom, mapping of reads exclusively to the *min$_{482}$-Su* precursor included in PVX. The *x*-axis indicates the position on the corresponding RNA sequence (subgenomic RNA or *min$_{482}$-Su* precursor in top and bottom graphs, respectively) in nucleotides of the 5' end of the sequence plotted. The *y*-axis is the small RNA coverage in total number of reads for each nucleotide position. B) Mapping of reads to the *min$_{482}$-Su* precursor expressed from the *35S:min$_{482}$-Su* construct. Other details are as in A.

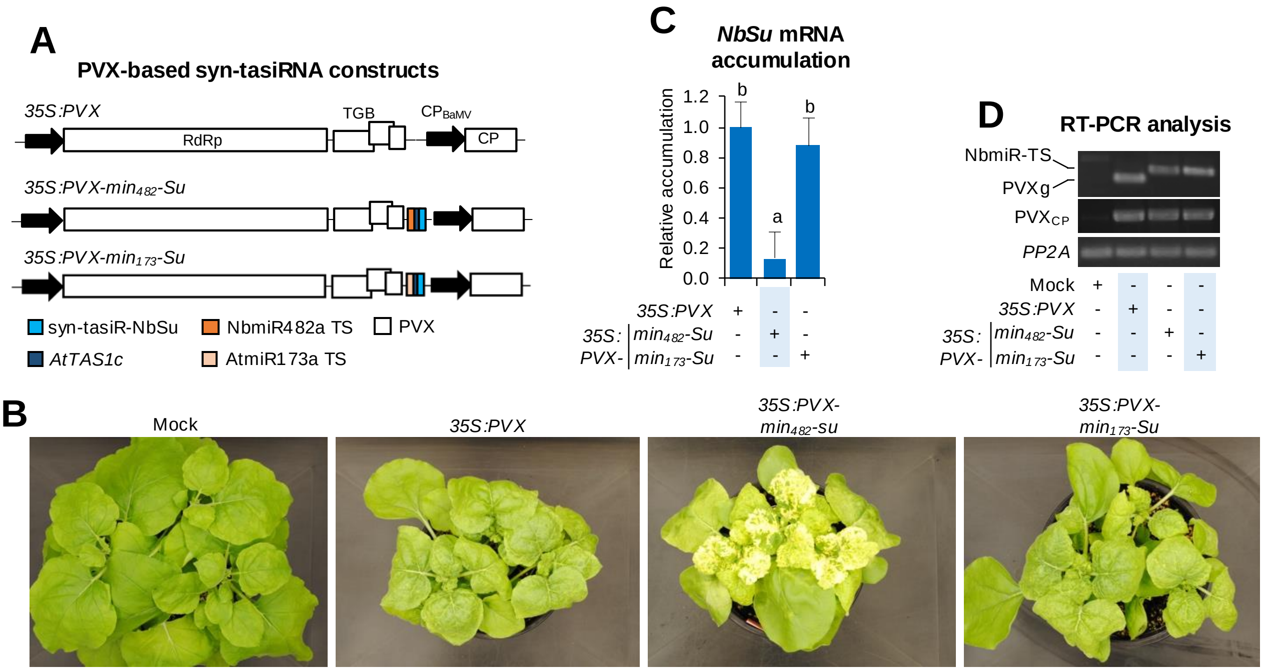

Figure S6. Functional analysis of potato virus X (PVX) constructs expressing syn-tasiR-NbSu from minimal precursors including endogenous or heterologous 22-nt miRNA target sites (TSs). (**A**) Diagram of PVX-based constructs. *AtTAS1c*, NbmiR482a TS, AtmiR173a TS and syn-tasiR-NbSu sequences are represented by dark blue, orange, light orange and light blue boxes, respectively. PVX ORFs and promoters are represented as white boxes and black arrows, respectively. RdRP, RNA-dependent RNA-polymerase; TGB, triple gene block; CP, coat protein; CPBaMV, Bamboo mosaic virus CP promoter. (**B**) Photos at 14 days post-agroinfiltration (dpa) of sets of three plants agroinoculated with the different constructs. (**C**) Target *NbSu* mRNA accumulation in RNA preparations from apical leaves collected at 14 days post-agroinfiltration (dpa) and analyzed individually (mock = 1.0 in all comparisons). Bars with the letter 'a' or 'b' indicate whether the mean values are significantly different from mock control or *35S:PVX-min*$_{482}$*-Su* samples, respectively ($P < 0.05$ in pairwise Student's t-test comparison). (**D**) RT-PCR detection of PVX and minimal precursors in apical leaves at 7 dpa. RT-PCR products corresponding to the *NbPP2A* and PVX vector controls are also shown (bottom). PVXg, band amplified from *35S:PVX* samples corresponding to the genomic region lacking a syn-tasiRNA precursor.

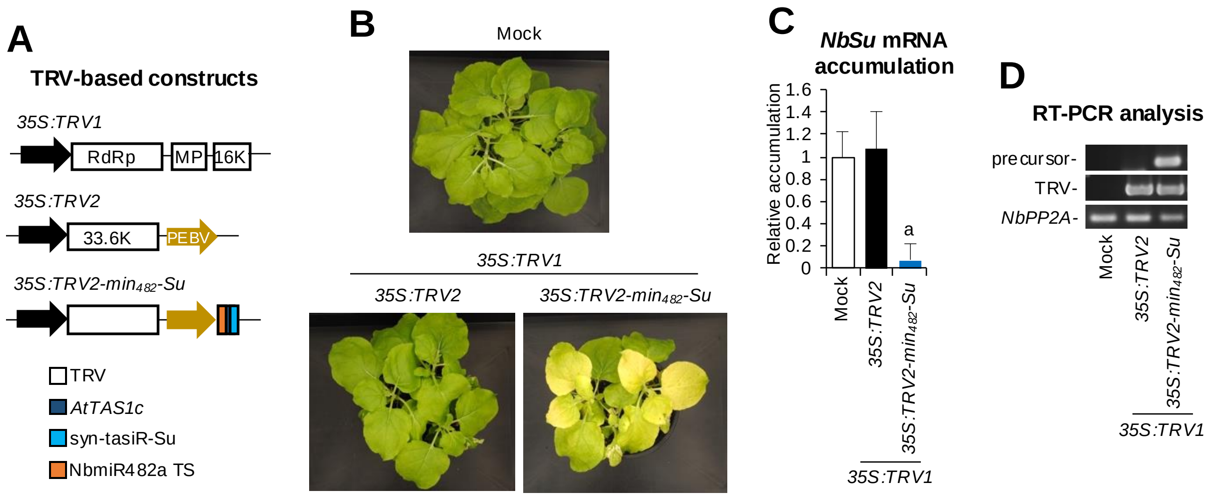

Figure S7. Functional analysis of tobacco rattle virus (TRV) constructs expressing syn-tasiR-Su from minimal syn-tasiRNA precursors in *N. benthamiana*. (**A**) Diagram of TRV-based constructs. *AtTAS1c*, NbmiR482a TS and syn-tasiR-Su sequences are represented by dark blue, orange and light blue boxes, respectively. TRV ORFs and 35S-based promoters are represented as white boxes and black arrows, respectively. RdRP, RNA-dependent RNA-polymerase; MP, movement protein; 16K, 16KDa protein; 33.6K, 33.6KDa protein; PEBV, pea early browning virus coat protein promoter. (**B**) Photos at 14 days post-agroinfiltration (dpa) of sets of three plants agroinoculated with the different constructs. (**C**) Target *NbSu* mRNA accumulation in RNA preparations from apical leaves collected at 14 dpa and analysed individually (mock = 1.0 in all comparisons). Bars with the letter 'a' indicate whether the mean values are significantly different from mock control samples (P < 0.05 in pairwise Student's *t*-test comparison). (**D**) RT-PCR detection of TRV and minimal precursors in apical leaves at 7 dpa. RT-PCR products corresponding to the *NbPP2A* are also shown.

Figure S7

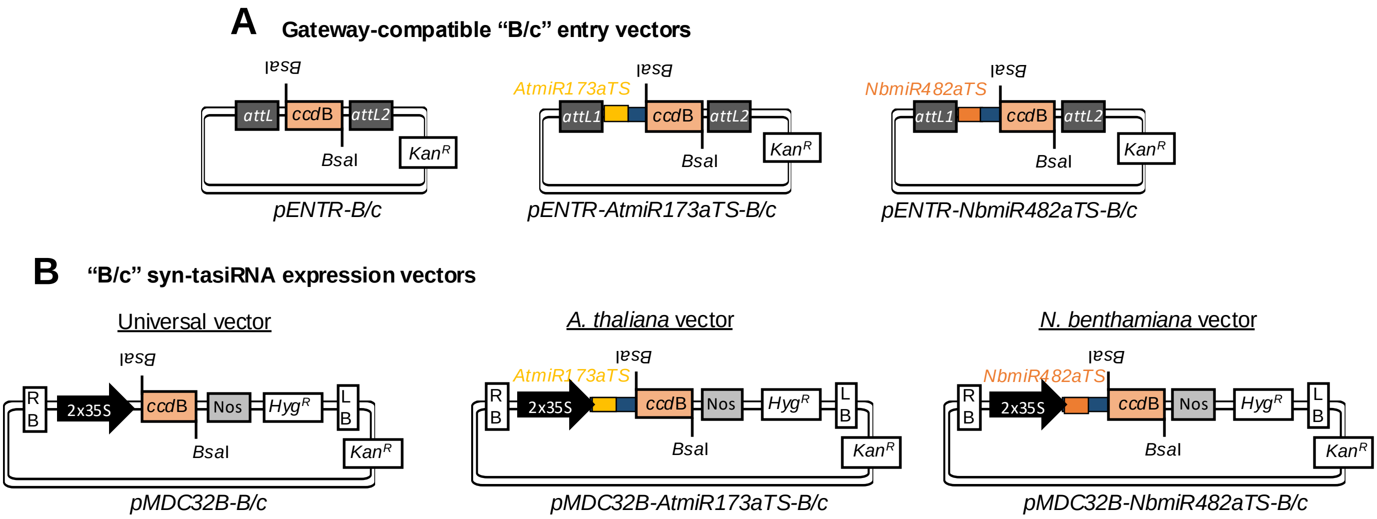

Figure S8. *B/c*-based vectors for direct cloning of syn-tasiRNAs downstream the AtmiR173a and NbmiR482a target sites (TSs), or the TS of choice. Sequences of AtmiR173a and NbmiR482a TSs are in yellow and orange, respectively. The spacer sequence derived from *AtTAS1c* is in blue. Top, diagram of the Gateway-compatible entry vectors. Bottom, diagram of binary vectors for in plant expression of amiRNAs. RB: right border; 35S: Cauliflower mosaic virus promoter; *Bsa*I: *Bsa*I recognition site, *ccd*B: gene encoding the gyrase toxin; LB: left border; attL1 and attL2: GATEWAY recombination sites. Kan^R: kanamycin resistance gene; Hyg^R: hygromycin resistance gene.

Table S1. Name, sequence and use of DNA oligonucleotides used in this study.

Name	Sequence	Type*	Construct/Aim
AC-55	AGGGGCCATGCTAATCTTCTC	ssDNA	Probe for U6 detection
AC-157	GGCCTCTTCCTTTATAACCAA	ssDNA	Probe for syn-tasiR-FT detection
AC-158	AGGGATTTCCGTGACACTTAA	ssDNA	Probe for amiR-CH42 detection
AC-159	AAAAATGGCTGAGGCTGATGA	ssDNA	qPCR amplification of *AtACT2* mRNA
AC-160	GAAAAACAGCCCTGGGAGC	ssDNA	
AC-163	CATGCACAAGTAGGGACGGTT	ssDNA	qPCR amplification of*AtCH42* mRNA
AC-164	GTCACGGAAATCCTTTGGGTT	ssDNA	
AC-169	TGGAACAACCTTTGGCAATG	ssDNA	qPCR amplification of *AtFT* mRNA
AC-170	CGACACGATGAATTCCTGCA	ssDNA	
AC-355	GACCCTGATGTTGATGTTCGCT	ssDNA	qPCR amplification of *NbSu* mRNA
AC-356	GAGGGATTTGAAGAGAGATTTC	ssDNA	
AC-365	GACCCTGATGTTGATGTTCGCT	ssDNA	PCR&qPCR amplification of *NbPP2A* mRNA
AC-366	GAGGGATTTGAAGAGAGATTTC	ssDNA	
AC-416	A+GGA+CAC+AAT+CAC+GTC+TTA+CA	ssLNA	Probe for amiR-TSWV/syn-tasiR-TSWV-1 detection
AC-417	G+CGG+GAA+GTC+CAC+CAC+GGT+TA	ssLNA	Probe for syn-tasiR-Su detection
AC-505	CCCCATACCACAACAAAACAATGCTGTTTCATCTCG	ssDNA	*35S:TAS$_{482}$-Su*
AC-507	TGTAAATGTTTAACAAAACAATGCTGTTTCATCTCG	ssDNA	*35S:TAS$_{6019}$-Su*
AC-631	GGAGTCGGGAATAGACCATTTATGTATGACTCCCGG	ssDNA	*35S:TAS$_{482}$-Su*
AC-632	AGTCACCTGTATAGACCATTTATGTATGACTCCCGG	ssDNA	*35S:TAS$_{6019}$-Su*
AC-639	CACCGTGGTATGGGGGGAGTCGGGAATAGACCATTTATCTTGTAACGCGCTTTCCCAG	ssDNA	*35S:min$_{482}$-GUS$_{Nb}$*
AC-640	CTGGGAAAGCGCGTTACAAGATAAATGGTCTATTCCCGACTCCCCCCATACCACGGTG	ssDNA	
AC-641	CACCGTGGTATGGGGGGAGTCGGGAATAGACCATTTATGTATGACTCCCGGAATTCCA	ssDNA	*35S:min$_{482}$-Su*
AC-642	TGGAATTCCGGGAGTCATACATAAATGGTCTATTCCCGACTCCCCCCATACCACGGTG	ssDNA	
AC-643	CACCAAACATTTACAAGTCACCTGTATAGACCATTTATCTTGTAACGCGCTTTCCCAG	ssDNA	*35S:min$_{6019}$-GUS$_{Nb}$*
AC-644	CTGGGAAAGCGCGTTACAAGATAAATGGTCTATACAGGTGACTTGTAAATGTTTGGTG	ssDNA	
AC-645	CACCAAACATTTACAAGTCACCTGTATAGACCATTTATGTATGACTCCCGGAATTCCA	ssDNA	*35S:min$_{6019}$-Su*
AC-646	TGGAATTCCGGGAGTCATACATAAATGGTCTATACAGGTGACTTGTAAATGTTTGGTG	ssDNA	
AC-650	GAGGTCAGCACCAGCTAGCAGTAGAGAAGAATCTGTA	ssDNA	*35S:PVX-amiR-GUS$_{Nb}$*, *35S:PVX-amiR-TSWV*
AC-654	GGGAATCAATCACAGTGTTGGC	ssDNA	syn-tasiRNA precursors detection
AC-655	GCTACTATGGCACGGGCTGTAC	ssDNA	
AC-657	ATGTCAGGCCTGTTCACTATCC	ssDNA	PVX diagnostic
AC-658	TGGTGGTGGTAGAGTGACAAC	ssDNA	
AC-663	GGGAAACTTAACAAACCCTAGTAAGAAGAGCCAA	ssDNA	*35S:PVX-amiR-GUS$_{Nb}$*, *35S:PVX-amiR-TSWV*
AC-666	AGAGGTCAGCACCAGCTAGCGTGGTATGGGGGGAGTCGGGAATAGACCATTTATGTATGACTCCCGGAATTCCAAGGGTTTGTTAAGTTTCCCT	dsDNA	*35S:PVX-min$_{482}$-GUS$_{Nb}$(x4)*
AC-667	CACTTACCCGAGTTAACGCCGTGGTATGGGGGGAGTCGGGAATAGACCATTTATGTATGACTCCCGGAATTCCAATGTCCCGAAGACATTAAAC	dsDNA	*35S:TRV2-min$_{482}$-Su*
AC-674	CACCTGTAAGAGACCGGTCTCACATT	ssDNA	*pENTR-BB, pENTR-B/c*
AC-675	AATGTGAGACCGGTCTCTTACAGGTG	ssDNA	*pMDC32B-BB, pMDC32B-B/c*

AC-676	CACCGTGATTTTTCTCTACAAGCGAATAGACCATTTATGCGCTTGCTGAGTTTCCCCC	ssDNA	*35S:min-GUS*$_{At}$
AC-677	GGGGGAAACTCAGCAAGCGCATAAATGGTCTATTCGCTTGTAGAGAAAAATCACGGTG	ssDNA	
AC-678	CACCGTGATTTTTCTCTACAAGCGAATAGACCATTTATTAAGTGTCACGGAAATCCCT	ssDNA	*35S:min-CH42*
AC-679	AGGGATTTCCGTGACACTTAATAAATGGTCTATTCGCTTGTAGAGAAAAATCACGGTG	ssDNA	
AC-680	CACCGTGATTTTTCTCTACAAGCGAATAGACCATTTATTGGTTATAAAGGAAGAGGCC	ssDNA	*35S:min-FT*
AC-681	GGCCTCTTCCTTTATAACCAATAAATGGTCTATTCGCTTGTAGAGAAAAATCACGGTG	ssDNA	
AC-682	CACCGTGATTTTTCTCTACAAGCGAATAGACCATTTATTGGTTATAAAGGAAGAGGCCTCCCATTCGATACTGCTCGCC	ssDNA	*35S:min-FT-TRY*
AC-683	GGCGAGCAGTATCGAATGGGAGGCCTCTTCCTTTATAACCAATAAATGGTCTATTCGCTTGTAGAGAAAAATCACGGTG	ssDNA	
AC-684	CACCGTGATTTTTCTCTACAAGCGAATAGACCATTTATCCCATTCGATACTGCTCGCCTTGGTTATAAAGGAAGAGGCC	ssDNA	*35S:minTRY-FT*
AC-685	GGCCTCTTCCTTTATAACCAAGGCGAGCAGTATCGAATGGGATAAATGGTCTATTCGCTTGTAGAGAAAAATCACGGTG	ssDNA	
AC-712	AGAGGTCAGCACCAGCTAGCAAACCTAAACCTAAACGG	ssDNA	*35S:PVX-TAS*$_{482}$*-Su*
AC-713	AGGGAAACTTAACAAACCCTATTTCACTTTACGATGTGG	ssDNA	
AC-716	TGTAGTGGTATGGGGGGAGTCGGGAATAGACCATTTATGTATGACTCCCGGAATTCCA	ssDNA	*35S:min*$_{482}$*-Su*
AC-717	AATGTGGAATTCCGGGAGTCATACATAAATGGTCTATTCCCGACTCCCCCCATACCAC	ssDNA	
AC-719	TGTAGTGCTCACTCTCTTCTGTCATAGACCATTTATGTATGACTCCCGGAATTCCA	ssDNA	*35S:min*$_{156}$*-Su*
AC-720	AATGTGGAATTCCGGGAGTCATACATAAATGGTCTATGACAGAAGAGAGTGAGCAC	ssDNA	
AC-721	TGTAGTGATTTTTCTCTACAAGCGAATAGACCATTTATGTATGACTCCCGGAATTCCA	ssDNA	*35S:min*$_{173}$*-Su*
AC-722	AATGTGGAATTCCGGGAGTCATACATAAATGGTCTATTCGCTTGTAGAGAAAAATCAC	ssDNA	
AC-900	TGTAGTGGTATGGGGGGAGTCGGGAATAGACCATTTAAGAGACCGGTCTCATCGG	ssDNA	*pENTR-NbmiR482aTS-B/c*<br>*pMDC32B-NbmiR482aTS-B/c*
AC-901	AATGCCGATGAGACCGGTCTCTTAAATGGTCTATTCCCGACTCCCCCCATACCAC	ssDNA	
AC-902	TGTAGGCATGGGCGGTGTAGGCAAGATAGACCATTTAAGAGACCGGTCTCATCGG	ssDNA	*pENTR-NbmiR6019a/bTS-B/c*<br>*pMDC32B-NbmiR6019a/bTS-B/c*
AC-903	AATGCCGATGAGACCGGTCTCTTAAATGGTCTATCTTGCCTACACCGCCCATGCC	ssDNA	
AC-935	TTTATGTAAGACGTGATTGTGTCCTTATCAGCTCTGGGTGAATCGGTTGGTATAGTGGGGCATACCGTCAGAGTGCACAATCCATCTT	ssDNA	*35S:min*$_{482}$*-TSWV(x4)*
AC-936	CCGAAAGATGGATTGTGCACTCTGACGGTATGCCCCACTATACCAACCGATTCACCCAGAGCTGATAAGGACACAATCACGTCTTACA	ssDNA	
AC-987	AGAGGTCAGCACCAGCTAGCGTGGTATGGGGGGAGTCGGGAATAGACCATTTATATTGACCCACACTTTGCCGATAACCTTCACCCGGTTGCCACTATTGACCCACACTTTGCCGATAACCTTCACCCGGTTGCCACAGGGTTTGTTAAGTTTCCCT	dsDNA	*35S:PVX-min*$_{482}$*-GUS*$_{Nb}$*(x4)*
AC-988	AGAGGTCAGCACCAGCTAGCGTGGTATGGGGGGAGTCGGGAA	ssDNA	*35S:PVX-NbmiR482aTS-TSWV(x4)*
AC-989	AGGGAAACTTAACAAACCCTAAGATGGATTGTGCACTCTGA	ssDNA	
AC-1093	TGTAGTGATTTTTCTCTACAAGCGAATAGACCATTTAAGAGACCGGTCTCATCGG	ssDNA	*pENTR-AtmiR173aTS-B/c*<br>*pMDC32B-AtmiR173aTS-B/c*

AC-1094	AATGCCGATGAGACCGGTCTCTTAAATGGT CTATTCGCTTGTAGAGAAAAATCAC	ssDNA	

*ssDNA: single-stranded DNA; dsDNA: double-stranded DNA; LNA: locked nucleic acid.

Table S2. Main features of DNA constructs functionally analyzed *in vivo* in this study.

**Construct**	**Plant species tested**	**Viral vector**	**Precursor**	**22-nt miRNA TS**	**Expressed art-sRNA(s)**	**Predicted target**	**Predicted art-sRNA off-targets (OT)**[1]	**Reference**
35S:min-CH42	*A. thaliana*	-	minimal	AtmiR173a	syn-tasiR-CH42	*AtCH42*	N/A	This work
35S:min-FT	*A. thaliana*	-	minimal	AtmiR173a	syn-tasiR-FT	*AtFT*	N/A	This work
35S:min-FT-TRY	*A. thaliana*	-	minimal	AtmiR173a	syn-tasiR-FT<br>syn-tasiR-TRY	*AtFT*<br>*AtTRY*	N/A<br>N/A	This work
35S:min-GUS$_{At}$	*A. thaliana*	-	minimal	AtmiR173a	syn-tasiR-GUS$_{At}$	*GUS*	No OT in *A. thaliana*	This work
35S:min-TRY-FT	*A. thaliana*	-	minimal	AtmiR173a	syn-tasiR-TRY<br>syn-tasiR-FT	*AtTRY*<br>*AtFT*	N/A<br>N/A	This work
35S:min$_{156}$*-Su*	*N. benthamiana*	-	minimal	NbmiR156a	syn-tasiR-Su	*NbSu*	No OT in *N. benthamiana*	This work
35S:min$_{173}$*-Su*	*N. benthamiana*	-	minimal	AtmiR173a	syn-tasiR-Su	*NbSu*	No OT in *N. benthamiana*	This work
35S:min$_{482}$*-GUS*$_{Nb}$	*N. benthamiana*	-	minimal	NbmiR482a	syn-tasiR-GUS$_{Nb}$	*GUS*	No OT in *N. benthamiana*	This work
35S:min$_{482}$*-Su*	*N. benthamiana*	-	minimal	NbmiR482a	syn-tasiR-Su	*NbSu*	No OT in *N. benthamiana*	This work
35S:min$_{482}$*-TSWV(x4)*	*N. benthamiana*	-	minimal	NbmiR482a	syn-tasiR-TSWV-1<br>syn-tasiR-TSWV-2<br>syn-tasiR-TSWV-3<br>syn-tasiR-TSWV-4	TSWV L<br>TSWV M<br>TSWV L<br>TSWV M	No OT in *S. lycopersicum*	This work
35S:min$_{6019}$*-GUS*$_{Nb}$	*N. benthamiana*		minimal	NbmiR482a	syn-tasiR-GUS$_{Nb}$	*GUS*	No OT in *N. benthamiana*	This work
35S:min$_{6019}$*-Su*	*N. benthamiana*		minimal	NbmiR482a	syn-tasiR-GUS$_{Nb}$	*GUS*	No OT in *N. benthamiana*	This work
35S:PVX-amiR-GUS$_{Nb}$	*N. benthamiana*	PVX	*MIR390a*[3]	-	amiR-GUS$_{Nb}$	*GUS*	No OT in *N. benthamiana*	This work
35S:PVX-amiR-TSWV	*N. benthamiana*	PVX	*MIR390a*	-	amiR-TSWV-1	TSWV L	No OT in *S. lycopersicum*	This work
35S:PVX-min$_{482}$*-GUS*$_{Nb}$(x4)	*N. benthamiana*	PVX	minimal	NbmiR482a	syn-tasiR-GUS$_{Nb}$-1<br>syn-tasiR-GUS$_{Nb}$-2<br>syn-tasiR-GUS$_{Nb}$-1<br>syn-tasiR-GUS$_{Nb}$-2	*GUS*<br>*GUS*<br>*GUS*<br>*GUS*	No OT in *N. benthamiana*	This work
35S:PVX-min$_{482}$*-Su*	*N. benthamiana*	PVX	minimal	NbmiR482a	syn-tasiR-Su	*NbSu*	No OT in *N. benthamiana*	This work
35S:PVX-min$_{482}$*-TSWV*(x4)	*N. benthamiana*	PVX	minimal	NbmiR482a	syn-tasiR-TSWV-1<br>syn-tasiR-TSWV-2<br>syn-tasiR-TSWV-3<br>syn-tasiR-TSWV-4	TSWV L<br>TSWV M<br>TSWV L<br>TSWV M	No OT in *N. benthamiana*	This work
35S-TAS-CH42	*A. thaliana*	-	*AtTAS1c*	AtmiR173a	syn-tasiR-CH42	*AtCH42*	N/A	(18)[4]
35S-TAS-FT	*A. thaliana*	-	*AtTAS1c*	AtmiR173a	syn-tasiR-FT	*AtFT*	N/A[2]	(18)
35S-TAS-GUS$_{At}$	*A. thaliana*	-	*AtTAS1c*	AtmiR173a	syn-tasiR-GUS$_{At}$	*GUS*	No OT in *A. thaliana*	(18)
35S-TAS-GUS$_{Nb}$*/MIR173*	*A. thaliana*	-	*AtTAS1c*	AtmiR173a	syn-tasiR-GUS$_{At}$<br>miR173a	*GUS*<br>*AtTAS1c*	No OT in *N. benthamiana*<br>-	(18)
35S:TAS-FT-TRY	*A. thaliana*	-	*AtTAS1c*	AtmiR173a	syn-tasiR-FT<br>syn-tasiR-TRY	*AtFT*<br>*AtTRY*	N/A<br>N/A	(18)
35S:TAS-Su/MIR173	*A. thaliana*	-	*AtTAS1c*	AtmiR173a	syn-tasiR-Su<br>miR173	*NbSu*<br>*AtTAS1c*	No OT in<br>N/A	(18)
35S:TAS-TRY-FT	*A. thaliana*	-	*AtTAS1c*	AtmiR173a	syn-tasiR-TRY	*AtTRY*	N/A	(18)

					syn-tasiR-FT	*AtFT*	N/A	
35S:TAS482-Su	*N. benthamiana*	-	*AtTAS1c*	NbmiR482a	syn-tasiR-Su	*NbSu*	No OT in *N. benthamiana*	This work
35S:TAS6019-Su	*N. benthamiana*	-	*AtTAS1c*	NbmiR482a	syn-tasiR-Su	*NbSu*	No OT in *N. benthamiana*	This work
35S-TRV2-min482-Su	*N. benthamiana*	TRV	minimal	NbmiR482a	syn-tasiR-Su	*NbSu*	No OT in *N. benthamiana*	This work

[1] "No OT" means that no off-targets were dected by a target prediction tool used during the art-sRNA design.

[2] N/C: means "not considered", that is no target prediction tool was used during the art-sRNA design.

[3] *MIR390a* refers here to the foldback, not the full-length precursor.

[4] (18): López-Dolz,L., Spada,M., Daròs,J.-A. and Carbonell,A. (2020) Fine-tune control of targeted RNAi efficacy by plant artificial small RNAs. Nucleic Acids Res, 48, 6234–6250.

Table S3: Phenotypic penetrance of syn-tasiRNAs expressed in *A. thaliana* Col-0 T1 transgenic plants for silencing *AtFT*.

**Construct**	**T1 analyzed**	**Phenotypic penetrance**[a]
35S:TAS-GUS$_{At}$	58	0%
35S:TAS-FT	65	100%
35S:min-FT	53	0%
35S:min-GUS$_{At}$	64	100%

[a] The FT phenotype was defined as a higher 'days to flowering' value when compared to the average 'days to flowering' value of the *35S:TAS-GUS*$_{At}$ and *35S:min-GUS*$_{At}$ control sets.

Table S4: Phenotypic penetrance of syn-tasiRNAs expressed in *A. thaliana* Col-0 T1 transgenic plants for silencing *AtCH42*.

**Construct**	**T1 analyzed**	**Phenotypic penetrance**[a]
35S:TAS-GUS$_{At}$	70	0%
35S:TAS-CH42	402	78%<br>17.9% weak<br>23.6% intermediate<br>36.1 % severe
35S:min-GUS$_{At}$	134	0%
35S:min-CH42	389	51%<br>7.2% weak<br>19.3% intermediate<br>24.7 % severe

[a] Ch42 phenotype is scored in 10 days-old seedling and is considered 'weak', 'intermediate' or 'severe' if seedlings have >2 leaves, exactly 2 leaves or no leaves (only 2 cotyledons), respectively.

Table S5: Phenotypic penetrance of syn-tasiRNAs expressed in Arabidopsis Col-0 T1 transgenic plants for silencing *AtFT* and *AtTRY*.

**Construct**	**T1 analyzed**	**Phenotypic penetrance[a]**
35S:TAS-GUS$_{At}$	46	0% FT<br>0% TRY
35S:TAS-FT-TRY	16	100% FT<br>88% TRY
35S:TAS-TRY-FT	16	100% FT<br>82% TRY
35S:min-GUS$_{At}$	27	0% FT<br>0% TRY
35S:min-FT-TRY	34	100% FT<br>80% TRY
35S:min-TRY-FT	20	100% FT<br>72% TRY

[a] The FT phenotype was defined as a higher 'days to flowering' value when compared to the average 'days to flowering' value of the *35S:TAS-GUS*$_{At}$ control set.

The TRY phenotype was defined as a higher number of trichomes when compared to transformants of the *35S:TAS-GUS*$_{At}$ control set.

Text S1. Protocol to design and clone syn-tasiRNAs downstream the 3'D1[+] position in *Bsa*I/*ccd*B-based ('B/c') vectors *pENTR-B/c*, *pMDC32-B/c*, *pENTR-AtmiR173aTS-B/c*, *pMDC32B-AtmiR173aTS-B/c*, *pENTR-NbmiR482aTS-B/c* and *pMDC32B-NbmiR482aTS-B/c*.

1. Selection of the syn-tasiRNA sequence(s)

Use the Syn-tasiRNA Designer app from the P-SAMS webtool at http://p-sams.carringtonlab.org/syntasi/designer.

2. Design of syn-tasiRNA oligonucleotides for cloning

Next are described some designs for cloning two syn-tasiRNAs in tandem downstream the 3'D1[+] position.

2.1. Using your 22-nt miRNA target site of choice:

Use vectors *pENTR-B/c* or *pMDC32-B/c* and order the following oligos

-Forward oligonucleotide (79 b):

TGTA$X_1X_2X_3X_4X_5X_6X_7X_8X_9X_{10}X_{11}X_{12}X_{13}X_{14}X_{15}X_{16}X_{17}X_{18}X_{19}X_{20}X_{21}X_{22}$TAGACCATTTA$X_1X_2X_3X_4X_5X_6X_7X_8X_9X_{10}X_{11}X_{12}X_{13}X_{14}X_{15}X_{16}X_{17}X_{18}X_{19}X_{20}X_{21}$$X_1X_2X_3X_4X_5X_6X_7X_8X_9X_{10}X_{11}X_{12}X_{13}X_{14}X_{15}X_{16}X_{17}X_{18}X_{19}X_{20}X_{21}$

-Reverse oligonucleotide (79 b):

AATG$Y_{21}Y_{20}Y_{19}Y_{18}Y_{17}Y_{16}Y_{15}Y_{14}Y_{13}Y_{12}Y_{11}Y_{10}Y_9Y_8Y_7Y_6Y_5Y_4Y_3Y_2Y_1$$Y_{21}Y_{20}Y_{19}Y_{18}Y_{17}Y_{16}Y_{15}Y_{14}Y_{13}Y_{12}Y_{11}Y_{10}Y_9Y_8Y_7Y_6Y_5Y_4Y_3Y_2Y_1$TAAATGGTCTA$Y_{22}Y_{21}Y_{20}Y_{19}Y_{18}Y_{17}Y_{16}Y_{15}Y_{14}Y_{13}Y_{12}Y_{11}Y_{10}Y_9Y_8Y_7Y_6Y_5Y_4Y_3Y_2Y_1$

Where:

TAGACCATTTA=*AtTAS1c*-derived spacer sequence

$X_1X_2X_3X_4X_5X_6X_7X_8X_9X_{10}X_{11}X_{12}X_{13}X_{14}X_{15}X_{16}X_{17}X_{18}X_{19}X_{20}X_{21}X_{22}$=22-nt miRNA target site sequence

$X_1X_2X_3X_4X_5X_6X_7X_8X_9X_{10}X_{11}X_{12}X_{13}X_{14}X_{15}X_{16}X_{17}X_{18}X_{19}X_{20}X_{21}$=syn-tasiRNA-1 sequence

$X_1X_2X_3X_4X_5X_6X_7X_8X_9X_{10}X_{11}X_{12}X_{13}X_{14}X_{15}X_{16}X_{17}X_{18}X_{19}X_{20}X_{21}$=syn-tasiRNA-2 sequence

$Y_{21}Y_{20}Y_{19}Y_{18}Y_{17}Y_{16}Y_{15}Y_{14}Y_{13}Y_{12}Y_{11}Y_{10}Y_9Y_8Y_7Y_6Y_5Y_4Y_3Y_2Y_1$=syn-tasiRNA-1 reverse-complement sequence

$Y_{21}Y_{20}Y_{19}Y_{18}Y_{17}Y_{16}Y_{15}Y_{14}Y_{13}Y_{12}Y_{11}Y_{10}Y_9Y_8Y_7Y_6Y_5Y_4Y_3Y_2Y_1$=syn-tasiRNA-2 reverse-complement sequence

TAAATGGTCTA=*AtTAS1c*-derived reverse-complement spacer sequence

$Y_{22}Y_{21}Y_{20}Y_{19}Y_{18}Y_{17}Y_{16}Y_{15}Y_{14}Y_{13}Y_{12}Y_{11}Y_{10}Y_9Y_8Y_7Y_6Y_5Y_4Y_3Y_2Y_1$=22-nt miRNA target site reverse-complement sequence

Example

The sequences of the two oligonucleotides to clone syn-tasiRNAs 'syn-tasiR-TRY' (**TCCCATTCGATACTGCTCGCC**) and 'syn-tasiR-Ft' (**TTGGTTATAAAGGAAGAGGCC**) in positions 3'D2[+] and 3'D3[+], respectively, of a minimal precursors including the *AtmiR173aTS* (**GTGATTTTTCTCTACAAGCGAA**) are:

-Forward oligonucleotide (79b):

TGTAGTGATTTTTCTCTACAAGCGAATAGACCATTTA**TCCCATTCGATACTGCTCGCCTTGGTTATAA AGGAAGAGGCC**

-Reverse oligonucleotide (79 b):

AATGGGCCTCTTCCTTTATAACCAAGGCGAGCAGTATCGAATGGGATAAATGGTCTA**TTCGCTTGTAG AGAAAAATCAC**

2.2. Using AtmiR173a or NbmiR482a miRNAs target site:

Use vectors *pENTR-AtmiR173aTS-B/c*, *pMDC32-AtmiR173aTS-B/c*, *pENTR-NbmiR482aTS-B/c* or *pMDC32-AtmiR482aTS-B/c* and order the following oligos:

-Forward oligonucleotide (46 b):

TTTA$X_1X_2X_3X_4X_5X_6X_7X_8X_9X_{10}X_{11}X_{12}X_{13}X_{14}X_{15}X_{16}X_{17}X_{18}X_{19}X_{20}X_{21}X_1X_2X_3X_4X_5X_6X_7X_8X_9X_{10}X_{11}X_{12}$ $X_{13}X_{14}X_{15}X_{16}X_{17}X_{18}X_{19}X_{20}X_{21}$

-Reverse oligonucleotide (46 b):

CCGA$Y_{21}Y_{20}Y_{19}Y_{18}Y_{17}Y_{16}Y_{15}Y_{14}Y_{13}Y_{12}Y_{11}Y_{10}Y_9Y_8Y_7Y_6Y_5Y_4Y_3Y_2Y_1Y_{21}Y_{20}Y_{19}Y_{18}Y_{17}$ $Y_{16}Y_{15}Y_{14}Y_{13}Y_{12}Y_{11}Y_{10}Y_9Y_8Y_7Y_6Y_5Y_4Y_3Y_2Y_1$

Where:

$X_1X_2X_3X_4X_5X_6X_7X_8X_9X_{10}X_{11}X_{12}X_{13}X_{14}X_{15}X_{16}X_{17}X_{18}X_{19}X_{20}X_{21}$=syn-tasiRNA-1 sequence

$X_1X_2X_3X_4X_5X_6X_7X_8X_9X_{10}X_{11}X_{12}X_{13}X_{14}X_{15}X_{16}X_{17}X_{18}X_{19}X_{20}X_{21}$=syn-tasiRNA-2 sequence

$Y_{21}Y_{20}Y_{19}Y_{18}Y_{17}Y_{16}Y_{15}Y_{14}Y_{13}Y_{12}Y_{11}Y_{10}Y_9Y_8Y_7Y_6Y_5Y_4Y_3Y_2Y_1$=syn-tasiRNA-1 reverse-complement sequence

$Y_{21}Y_{20}Y_{19}Y_{18}Y_{17}Y_{16}Y_{15}Y_{14}Y_{13}Y_{12}Y_{11}Y_{10}Y_9Y_8Y_7Y_6Y_5Y_4Y_3Y_2Y_1$=syn-tasiRNA-2 reverse-complement sequence

Example

The sequences of the two oligonucleotides to clone syn-tasiRNAs 'syn-tasiR-TRY' (**TCCCATTCGATACTGCTCGCC**) and 'syn-tasiR-Ft' (**TTGGTTATAAAGGAAGAGGCC**) in positions 3'D2[+] and 3'D3[+], respectively, of minimal precursors included in AtmiR173aTS- or NbmiR482aTS-based B/c vectors are:

-Forward oligonucleotide (46 b):

TTTATCCCATTCGATACTGCTCGCCTTGGTTATAAAGGAAGAGGCC

-Reverse oligonucleotide (46 b):

CCGAGGCCTCTTCCTTTATAACCAAGGCGAGCAGTATCGAATGGGA

3. Cloning of the syn-tasiRNA sequence(s) in B/c-based vectors

Notes:

-New available -B/c vectors are listed in Table I at the end of the section.

-B/c-*based vectors must be propagated in a* ccdB *resistant* E. coli *strain such as DB3.1.*

-Alternatively, BsaI *digestion of the B/c vector and subsequent ligation of the amiRNA oligonucleotide insert can be done in separate reactions*

3.1. Oligonucleotide annealing

-Dilute sense oligonucleotide and antisense oligonucleotide in sterile H_2O to a final concentration of 100 μM.

-Prepare Oligo Annealing Buffer:

60 mM Tris-HCl (pH 7.5)

500 mM NaCl

60 mM $MgCl_2$

10 mM DTT

Note: *Prepare 1 ml aliquots of Oligo Annealing Buffer and store at -20⁰C.*

-Assemble the annealing reaction in a PCR tube as described below:

Forward oligonucleotide (100 μM)	2 μL
Reverse oligonucleotide (100 μM)	2 μL
Oligo Annealing Buffer	46 μL
Total volume	50 μL

The final concentration of each oligonucleotide is 4 μM.

-Use a thermocycler to heat the annealing reaction 5 min at 94°C and then cool down (0.05ºC/sec) to 20°C.

-Dilute the annealed oligonucleotides just prior to assembling the digestion-ligation reaction as described below:

Annealed oligonucleotides	3 µL
dH_2O	37 µL
Total volume	40 µL

The final concentration of each oligonucleotide is 0.15 µM.

Note: *Do not store the diluted oligonucleotides.*

3.2. Digestion-ligation reaction

- Assemble the digestion-ligation reaction as described below:

B/c vector (x ug/uL)	Y µL (50 ng)
Diluted annealed oligonucleotides	1 µL
10x T4 DNA ligase buffer	1 µL
T4 DNA ligase (400 U/µL)	1 µL
*Bsa*I (10U/ µL, NEB)	1 µL
dH_2O	to 10 µL
Total volume	10 µL

Prepare a negative control reaction lacking *Bsa*I.

-Mix the reactions by pipetting. Incubate the reactions at room temperature for 5 minutes at 37°C.

3.3. *E.coli* transformation and analysis of transformants

-Transform 1-5 ul of the digestion-ligation reaction into an *E. coli* strain that doesn't have *ccd*B resistance (e.g. DH10B, TOP10, …) to do counter-selection.

-Pick two colonies/construct, grow LB-Kan (100 mg/ml) cultures and purify plasmids.

-Sequence with appropriate primers: M13-F (CCCAGTCACGACGTTGTAAAACGACGG) and

M13-R (CAGAGCTGCCAGGAAACAGCTATGACC) for *pENTR*-based vectors; attB1 (ACAAGTTTGTACAAAAAAGCAGGCT) and attB2 (ACCACTTTGTACAAGAAAGCTGGGT) primers for *pMDC32B*-based vectors).

Table I: *Bsa*I/*ccd*B-based ('B/c') vectors for direct cloning of syn-tasiRNAs downstream position 3'D1[+] in minimal precursor.

Vector	Small RNA expressed	Bacterial antibiotic resistance	Plant antibiotic resistance	GATEWAY use	Backbone	Promoter of syn-tasiRNA cassette	Terminator of syn-tasiRNA cassette	Plant species tested
pENTR-B/c	–	Kanamycin	–	Donor	*pENTR*	–	–	–
pMDC32B-B/c		Kanamycin<br>Hygromycin	Hygromycin	–	*pMDC32*	*CaMV* 2x35S	*Nos*	*A. thaliana*<br>*N. benthamiana*
pENTR-AtmiR173aTS-B/c	syn-tasiRNAs	Kanamycin	–	Donor	*pENTR*	–	–	–
pMDC32B-AtmiR173aTS-B/c	syn-tasiRNAs	Kanamycin<br>Hygromycin	Hygromycin	–	*pMDC32*	*CaMV* 2x35S	*Nos*	*A. thaliana*
pENTR-NbmiR482aTS-B/c	syn-tasiRNAs	Kanamycin	–	Donor	*pENTR*	–	–	–
pMDC32B-NbmiR482aTS-B/c	syn-tasiRNAs	Kanamycin<br>Hygromycin	Hygromycin	–	*pMDC32*	*CaMV* 2x35S	*Nos*	*N. benthamiana*

Text S2. Protocol to generate PVX-based syn-tasiRNA constructs.

1. Preparation of the dsDNA syn-tasiRNA insert

Design and order a dsDNA (eg. ultramer duplex in IDT) including the sequences of your syn-tasiRNA(s) (2 in the following example) following the 22-nt miRNA target site of interest, as follows:

agaggtcagcaccagctagc$X_1X_2X_3X_4X_5X_6X_7X_8X_9X_{10}X_{11}X_{12}X_{13}X_{14}X_{15}X_{16}X_{17}X_{18}X_{19}X_{20}X_{21}X_{22}$TAGAC
CATTTA$X_1X_2X_3X_4X_5X_6X_7X_8X_9X_{10}X_{11}X_{12}X_{13}X_{14}X_{15}X_{16}X_{17}X_{18}X_{19}X_{20}X_{21}X_1X_2X_3X_4X_5X_6X_7X_8X_9X_{10}X_{11}X_{12}X_{13}X_{14}X_{15}X_{16}X_{17}X_{18}X_{19}X_{20}X_{21}$agggtttgttaagtttccct

Where:

-X is a DNA base of the 22-nt miRNA target site sequence, and the subscript number is the base position

-X is a DNA base of the syn-tasiRNA-1 sequence, and the subscript number is the base position in the syn-tasiRNA* 21-mer

-X is a DNA base of the syn-tasiRNA-2 sequence, and the subscript number is the base position in the syn-tasiRNA 21-mer

-x is a DNA base of the PVX sequence, required for Gibson-based assembly

-X is a DNA base of the *AtTAS1c* sequence

Note that:

-In general, **X_1=T** and **X_1=T** for amiRNA association with AGO1.

Fragment #1 (syn-tasiRNA precursor) is ready.

2. Preparation of the vector

-Digest *pLB-PVX* with *Mlu*I.

-Gel purify the 9921 bp band corresonding to linearized plasmid.

-Quantify 1 ul in Nanodrop.

Fragment #2 (backbone vector) is ready.

3. Assembly

-Assemble the Gibbson reaction as described below:

Fragment 1 (dsDNA insert)[a]	
Fragment 2 (vector)[b,c,d]	
GeneArt Gibson Assembly HiFI Master Mix	5 µL
dH_2O	to 10 µL

Total volume 10 µL

[a]The optimal amount of vector is between 50-100 ng

[b]Insert/vector molar excess is between 2-3.

[c]Total DNA amount is between 0.02-0.5 pmol

[d]Mass to moles conversions can be calculated here:

http://nebiocalculator.neb.com/#!/ssdnaamt

-Incubate reactions at 50ºC for 1h.

-Clean up reactions with a column (e.g. Zymo Research)

-Transform 1-4 µL in *E. coli* DH5α

-Plate in L-Kan plates and incubate 16h at 37ºC

4. Clone verification

-Pick several colonies and grow in liquid LB-Kan 16h at 37ºC, and purify plasmids.

-Digest candidate clones with *Apa*I+*Xho*I

Good clones: 8595 + **1409** bp

Bad clones (empty *pLB-PVX*): 9921 bp + **1738** bp

-Confirm insert sequence by Sanger sequencing with forward and reverse oligos AC-654 (`GGGAATCAATCACAGTGTTGGC`) and/or AC-655 (`GCTACTATGGCACGGGCTGTAC`), respectively.

Text S3. DNA sequence in FASTA format of all precursors used to express art-sRNAs in plants.

1. *AtTAS1c*-based precursors

>TAS-GUS$_{At}$

```
AAACCTAAACCTAAACGGCTAAGCCCGACGTCAAATACCAAAAAGAGAAAAACAAGAGCGCCGTCAAGCTCTGCAAATACGATCTGTAAG
TCCATCTTAACACAAAAGTGAGATGGGTTCTTAGATCATGTTCCGCCGTTAGATCGAGTCATGGTCTTGTCTCATAGAAAGGTACTTTCG
TTTACTTCTTTTGAGTATCGAGTAGAGCGTCGTCTATAGTTAGTTTGAGATTGCGTTTGTCAGAAGTTAGGTTCAATGTCCCGGTCCAAT
TTTCACCAGCCATGTGTCAGTTTCGTTCCTTCCCGTCCTCTTCTTTGATTTCGTTGGGTTACGGATGTTTTCGAGATGAAACAGCATTGT
TTTGTTGTGATTTTTCTCTACAAGCGAATAGACCATTTATGCGCTTGCTGAGTTTCCCCCTCGGTGGATCTTAGAAAATTATTCTAAGTC
CAACATAGCGTATTCTAAGTTCAACATATCGACGAACTAGAAAAGACATTGGACATATTCCAGGATATGCAAAAGAAAACAATGAATATT
GTTTTGAATGTGTTCAAGTAAATGAGATTTTCAAGTCGTCTAAAGAACAGTTGCTAATACAGTTACTTATTTCAATAAATAATTGGTTCT
AATAATACAAAACATATTCGAGGATATGCAGAAAAAAAGATGTTTGTTATTTTGAAAAGCTTGAGTAGTTTCTCTCCGAGGTGTAGCGAA
GAAGCATCATCTACTTTGTAATGTAATTTTCTTTATGTTTTCACTTTGTAATTTTATTTGTGTTAATGTACCATGGCCGATATCGGTTTT
ATTGAAAGAAAATTTATGTTACTTCTGTTTTGGCTTTGCAATCAGTTATGCTAGTTTTCTTATACCCTTTCGTAAGCTTCCTAAGGAATC
GTTCATTGATTTCCACTGCTTCATTGTATATTAAAACTTTACAACTGTATCGACCATCATATAATTCTGGGTCAAGAGATGAAAATAGAA
CACCACATCGTAAAGTGAAAT
```

AtTAS1c
AtmiR173a TS
syn-tasiR-GUS$_{At}$

>TAS-FT

```
AAACCTAAACCTAAACGGCTAAGCCCGACGTCAAATACCAAAAAGAGAAAAACAAGAGCGCCGTCAAGCTCTGCAAATACGATCTGTAAG
TCCATCTTAACACAAAAGTGAGATGGGTTCTTAGATCATGTTCCGCCGTTAGATCGAGTCATGGTCTTGTCTCATAGAAAGGTACTTTCG
TTTACTTCTTTTGAGTATCGAGTAGAGCGTCGTCTATAGTTAGTTTGAGATTGCGTTTGTCAGAAGTTAGGTTCAATGTCCCGGTCCAAT
TTTCACCAGCCATGTGTCAGTTTCGTTCCTTCCCGTCCTCTTCTTTGATTTCGTTGGGTTACGGATGTTTTCGAGATGAAACAGCATTGT
TTTGTTGTGATTTTTCTCTACAAGCGAATAGACCATTTATTGGTTATAAAGGAAGAGGCCTCGGTGGATCTTAGAAAATTATTCTAAGTC
CAACATAGCGTATTCTAAGTTCAACATATCGACGAACTAGAAAAGACATTGGACATATTCCAGGATATGCAAAAGAAAACAATGAATATT
GTTTTGAATGTGTTCAAGTAAATGAGATTTTCAAGTCGTCTAAAGAACAGTTGCTAATACAGTTACTTATTTCAATAAATAATTGGTTCT
AATAATACAAAACATATTCGAGGATATGCAGAAAAAAAGATGTTTGTTATTTTGAAAAGCTTGAGTAGTTTCTCTCCGAGGTGTAGCGAA
GAAGCATCATCTACTTTGTAATGTAATTTTCTTTATGTTTTCACTTTGTAATTTTATTTGTGTTAATGTACCATGGCCGATATCGGTTTT
ATTGAAAGAAAATTTATGTTACTTCTGTTTTGGCTTTGCAATCAGTTATGCTAGTTTTCTTATACCCTTTCGTAAGCTTCCTAAGGAATC
GTTCATTGATTTCCACTGCTTCATTGTATATTAAAACTTTACAACTGTATCGACCATCATATAATTCTGGGTCAAGAGATGAAAATAGAA
CACCACATCGTAAAGTGAAAT
```

AtTAS1c
AtmiR173a TS
syn-tasiR-AtFT

>TAS-CH42

```
AAACCTAAACCTAAACGGCTAAGCCCGACGTCAAATACCAAAAAGAGAAAAACAAGAGCGCCGTCAAGCTCTGCAAATACGATCTGTAAG
TCCATCTTAACACAAAAGTGAGATGGGTTCTTAGATCATGTTCCGCCGTTAGATCGAGTCATGGTCTTGTCTCATAGAAAGGTACTTTCG
TTTACTTCTTTTGAGTATCGAGTAGAGCGTCGTCTATAGTTAGTTTGAGATTGCGTTTGTCAGAAGTTAGGTTCAATGTCCCGGTCCAAT
TTTCACCAGCCATGTGTCAGTTTCGTTCCTTCCCGTCCTCTTCTTTGATTTCGTTGGGTTACGGATGTTTTCGAGATGAAACAGCATTGT
TTTGTTGTGATTTTTCTCTACAAGCGAATAGACCATTTATTAAGTGTCACGGAAATCCCTTCGGTGGATCTTAGAAAATTATTCTAAGTC
CAACATAGCGTATTCTAAGTTCAACATATCGACGAACTAGAAAAGACATTGGACATATTCCAGGATATGCAAAAGAAAACAATGAATATT
GTTTTGAATGTGTTCAAGTAAATGAGATTTTCAAGTCGTCTAAAGAACAGTTGCTAATACAGTTACTTATTTCAATAAATAATTGGTTCT
AATAATACAAAACATATTCGAGGATATGCAGAAAAAAAGATGTTTGTTATTTTGAAAAGCTTGAGTAGTTTCTCTCCGAGGTGTAGCGAA
GAAGCATCATCTACTTTGTAATGTAATTTTCTTTATGTTTTCACTTTGTAATTTTATTTGTGTTAATGTACCATGGCCGATATCGGTTTT
ATTGAAAGAAAATTTATGTTACTTCTGTTTTGGCTTTGCAATCAGTTATGCTAGTTTTCTTATACCCTTTCGTAAGCTTCCTAAGGAATC
GTTCATTGATTTCCACTGCTTCATTGTATATTAAAACTTTACAACTGTATCGACCATCATATAATTCTGGGTCAAGAGATGAAAATAGAA
CACCACATCGTAAAGTGAAAT
```

AtTAS1c
AtmiR173a TS
syn-tasiR-CH42

>TAS-FT-TRY

AAACCTAAACCTAAACGGCTAAGCCCGACGTCAAATACCAAAAAGAGAAAAACAAGAGCGCCGTCAAGCTCTGCAAATACGATCTGTAAG
TCCATCTTAACACAAAAGTGAGATGGGTTCTTAGATCATGTTCCGCCGTTAGATCGAGTCATGGTCTTGTCTCATAGAAAGGTACTTTCG
TTTACTTCTTTTGAGTATCGAGTAGAGCGTCGTCTATAGTTAGTTTGAGATTGCGTTTGTCAGAAGTTAGGTTCAATGTCCCGGTCCAAT
TTTCACCAGCCATGTGTCAGTTTCGTTCCTTCCCGTCCTCTTCTTTGATTTCGTTGGGTTACGGATGTTTTCGAGATGAAACAGCATTGT
TTTGTTGTGATTTTTCTCTACAAGCGAATAGACCATTTATTGGTTATAAAGGAAGAGGCCTCCCATTCGATACTGCTCGCCTCGGTGGAT
CTTAGAAAATTATTCTAAGTCCAACATAGCGTATTCTAAGTTCAACATATCGACGAACTAGAAAAGACATTGGACATATTCCAGG
ATATGCAAAAGAAAACAATGAATATTGTTTTGAATGTGTTCAAGTAAATGAGATTTTCAAGTCGTCTAAAGAACAGTTGCTAATACAGTT
ACTTATTTCAATAAATAATTGGTTCTAATAATACAAAACATATTCGAGGATATGCAGAAAAAAAGATGTTTGTTATTTTGAAAAGCTTGA
GTAGTTTCTCTCCGAGGTGTAGCGAAGAAGCATCATCTACTTTGTAATGTAATTTTCTTTATGTTTTCACTTTGTAATTTTATTTGTGTT
AATGTACCATGGCCGATATCGGTTTTATTGAAAGAAAATTTATGTTACTTCTGTTTTGGCTTTGCAATCAGTTATGCTAGTTTTCTTATA
CCCTTTCGTAAGCTTCCTAAGGAATCGTTCATTGATTTCCACTGCTTCATTGTATATTAAAACTTTACAACTGTATCGACCATCATATAA
TTCTGGGTCAAGAGATGAAAATAGAACACCACATCGTAAAGTGAAAT

AtTAS1c
AtmiR173a TS
syn-tasiR-FT
syn-tasiR-TRY

>TAS-TRY-FT
AAACCTAAACCTAAACGGCTAAGCCCGACGTCAAATACCAAAAAGAGAAAAACAAGAGCGCCGTCAAGCTCTGCAAATACGATCTGTAAG
TCCATCTTAACACAAAAGTGAGATGGGTTCTTAGATCATGTTCCGCCGTTAGATCGAGTCATGGTCTTGTCTCATAGAAAGGTACTTTCG
TTTACTTCTTTTGAGTATCGAGTAGAGCGTCGTCTATAGTTAGTTTGAGATTGCGTTTGTCAGAAGTTAGGTTCAATGTCCCGGTCCAAT
TTTCACCAGCCATGTGTCAGTTTCGTTCCTTCCCGTCCTCTTCTTTGATTTCGTTGGGTTACGGATGTTTTCGAGATGAAACAGCATTGT
TTTGTTGTGATTTTTCTCTACAAGCGAATAGACCATTTATCCCATTCGATACTGCTCGCCTTGGTTATAAAGGAAGAGGCCTCGGTGGAT
CTTAGAAAATTATTCTAAGTCCAACATAGCGTATTCTAAGTTCAACATATCGACGAACTAGAAAAGACATTGGACATATTCCAGGATATG
CAAAAGAAAACAATGAATATTGTTTTGAATGTGTTCAAGTAAATGAGATTTTCAAGTCGTCTAAAGAACAGTTGCTAATACAGTTACTTA
TTTCAATAAATAATTGGTTCTAATAATACAAAACATATTCGAGGATATGCAGAAAAAAAGATGTTTGTTATTTTGAAAAGCTTGAGTAGT
TTCTCTCCGAGGTGTAGCGAAGAAGCATCATCTACTTTGTAATGTAATTTTCTTTATGTTTTCACTTTGTAATTTTATTTGTGTTAATGT
ACCATGGCCGATATCGGTTTTATTGAAAGAAAATTTATGTTACTTCTGTTTTGGCTTTGCAATCAGTTATGCTAGTTTTCTTATACCCTT
TCGTAAGCTTCCTAAGGAATCGTTCATTGATTTCCACTGCTTCATTGTATATTAAAACTTTACAACTGTATCGACCATCATATAATTCTG
GGTCAAGAGATGAAAATAGAACACCACATCGTAAAGTGAAAT

AtTAS1c
AtmiR173a TS
syn-tasiR-TRY
syn-tasiR-FT

>TAS-Su
AAACCTAAACCTAAACGGCTAAGCCCGACGTCAAATACCAAAAAGAGAAAAACAAGAGCGCCGTCAAGCTCTGCAAATACGATCTGTAAG
TCCATCTTAACACAAAAGTGAGATGGGTTCTTAGATCATGTTCCGCCGTTAGATCGAGTCATGGTCTTGTCTCATAGAAAGGTACTTTCG
TTTACTTCTTTTGAGTATCGAGTAGAGCGTCGTCTATAGTTAGTTTGAGATTGCGTTTGTCAGAAGTTAGGTTCAATGTCCCGGTCCAAT
TTTCACCAGCCATGTGTCAGTTTCGTTCCTTCCCGTCCTCTTCTTTGATTTCGTTGGGTTACGGATGTTTTCGAGATGAAACAGCATTGT
TTTGTTGTGATTTTTCTCTACAAGCGAATAGACCATTTATGTATGACTCCCGGAATTCCATCGGTGGATCTTAGAAAATTATTCTAAGTC
CAACATAGCGTATTCTAAGTTCAACATATCGACGAACTAGAAAAGACATTGGACATATTCCAGGATATGCAAAAGAAAACAATGAATATT
GTTTTGAATGTGTTCAAGTAAATGAGATTTTCAAGTCGTCTAAAGAACAGTTGCTAATACAGTTACTTATTTCAATAAATAATTGGTTCT
AATAATACAAAACATATTCGAGGATATGCAGAAAAAAAGATGTTTGTTATTTTGAAAAGCTTGAGTAGTTTCTCTCCGAGGTGTAGCGAA
GAAGCATCATCTACTTTGTAATGTAATTTTCTTTATGTTTTCACTTTGTAATTTTATTTGTGTTAATGTACCATGGCCGATATCGGTTTT
ATTGAAAGAAAATTTATGTTACTTCTGTTTTGGCTTTGCAATCAGTTATGCTAGTTTTCTTATACCCTTTCGTAAGCTTCCTAAGGAATC
GTTCATTGATTTCCACTGCTTCATTGTATATTAAAACTTTACAACTGTATCGACCATCATATAATTCTGGGTCAAGAGATGAAAATAGAA
CACCACATCGTAAAGTGAAAT

AtTAS1c
AtmiR173a TS
syn-tasiR-Su

2. Minimal syn-tasiRNA precursors

>min-GUS$_{At}$

GTGATTTTTCTCTACAAGCGAATAGACCATTTATGCGCTTGCTGAGTTTCCCCC

AtTAS1c
AtmiR173a TS
syn-tasiR-GUS$_{At}$

>min-FT

GTGATTTTTCTCTACAAGCGAATAGACCATTTATTGGTTATAAAGGAAGAGGCC

AtTAS1c
AtmiR173a TS
syn-tasiR-AtFT

>min-CH42

GTGATTTTTCTCTACAAGCGAATAGACCATTTATTAAGTGTCACGGAAATCCCT

AtTAS1c
AtmiR173a TS
syn-tasiR-CH42

>min-FT-TRY

GTGATTTTTCTCTACAAGCGAATAGACCATTTATTGGTTATAAAGGAAGAGGCCTCCCATTCGATACTGCTCGCC

AtTAS1c
AtmiR173a TS
syn-tasiR-FT
syn-tasiR-TRY

>min-TRY-FT

GTGATTTTTCTCTACAAGCGAATAGACCATTTATCCCATTCGATACTGCTCGCCTTGGTTATAAAGGAAGAGGCC

AtTAS1c
AtmiR173a TS
syn-tasiR-TRY
syn-tasiR-FT

>min$_{173}$-Su

GTGATTTTTCTCTACAAGCGAATAGACCATTTATGTATGACTCCCGGAATTCCA

AtTAS1c
AtmiR173a TS
syn-tasiR-Su

>min$_{173}$-TSWV(x4)

GTGATTTTTCTCTACAAGCGAATAGACCATTTATGTAAGACGTGATTGTGTCCTTATCAGCTCTGGGTGAATCGGTTGGTATAG
TGGGGCATACCGTCAGAGTGCACAATCCATCTT

AtTAS1c
AtmiR173a TS
syn-tasiR-TSWV-1
syn-tasiR-TSWV-2
syn-tasiR-TSWV-3
syn-tasiR-TSWV-4

>min$_{482}$-Su

GTGGTATGGGGGGAGTCGGGAATAGACCATTTATGTATGACTCCCGGAATTCCA

AtTAS1c
NbmiR482a TS
syn-tasiR-Su

>min$_{482}$-GUS$_{Nb}$ (x4)
GTGGTATGGGGGGAGTCGGGAATAGACCATTTATATTGACCCACACTTTGCCGATAACCTTCACCCGGTTGCCACTATTGACCC
ACACTTTGCCGATAACCTTCACCCGGTTGCCAC

AtTAS1c
NbmiR482a TS
syn-tasiR-GUS$_{Nb}$-1
syn-tasiR-GUS$_{Nb}$-2

>min$_{482}$-TSWV (x4)
GTGGTATGGGGGGAGTCGGGAATAGACCATTTATGTAAGACGTGATTGTGTCCTTATCAGCTCTGGGTGAATCGGTTGGTATAG
TGGGGCATACCGTCAGAGTGCACAATCCATCTT

AtTAS1c
NbmiR482a TS
syn-tasiR-TSWV-1
syn-tasiR-TSWV-2
syn-tasiR-TSWV-3
syn-tasiR-TSWV-4

>min$_{6019}$-Su
AAACATTTACAAGTCACCTGTATAGACCATTTATGTATGACTCCCGGAATTCCA

AtTAS1c
NbmiR6019a/b TS
syn-tasiR-Su

>min$_{156}$-Su
GTGCTCACTCTCTTCTGTCATAGACCATTTATGTATGACTCCCGGAATTCCA

AtTAS1c
NbmiR156a TS
syn-tasiR-Su

>min$_{173}$-Su
GTGATTTTTCTCTACAAGCGAATAGACCATTTATGTATGACTCCCGGAATTCCA

AtTAS1c
AtmiR173a TS
syn-tasiR-Su

3. amiRNA precursors

>amiR-GUS$_{Nb}$
GTAGAGAAGAATCTGTATATTGACCCACACTTTGCCGAATGATGATCACATTCGTTATCTATTTTTTAGGCAAAGTTTGGGTCAATACAt
tggctcttcttact

AtMIR390a
amiR-GUS$_{Nb}$
amiR-GUS$_{Nb}$*

>amiR-TSWV
GTAGAGAAGAATCTGTATGTAAGACGTGATTGTGTCCTATGATGATCACATTCGTTATCTATTTTTTAGGACACAATAACGTCTTACACA
ttggctcttcttact

AtMIR390a
amiR-TSWV
amiR-TSWV*

Text S4. DNA sequence of *Bsa*I-*ccd*B-based (B/c) vectors used for direct cloning of syn-tasiRNAs.

>pENTR-B/c (4049 bp)

CTTTCCTGCGTTATCCCCTGATTCTGTGGATAACCGTATTACCGCCTTTGAGTGAGCTGATACCGCTCGCCGCAGCCGAACGACCGAGCG
CAGCGAGTCAGTGAGCGAGGAAGCGGAAGAGCGCCCAATACGCAAACCGCCTCTCCCCGCGCGTTGGCCGATTCATTAATGCAGCTGGCA
CGACAGGTTTCCCGACTGGAAAGCGGGCAGTGAGCGCAACGCAATTAATACGCGTACCGCTAGCCAGGAAGAGTTTGTAGAAACGCAAAA
AGGCCATCCGTCAGGATGGCCTTCTGCTTAGTTTGATGCCTGGCAGTTTATGGCGGGCGTCCTGCCCGCCACCCTCCGGGCCGTTGCTTC
ACAACGTTCAAATCCGCTCCCGGCGGATTTGTCCTACTCAGGAGAGCGTTCACCGACAAACAACAGATAAAACGAAAGGCCCAGTCTTCC
GACTGAGCCTTTCGTTTTATTTGATGCCTGGCAGTTCCCTACTCTCGCGTTAACGCTAGCATGGATGTTTTCCCAGTCACGACGTTGTAA
AACGACGGCCAGTCTTAAGCTCGGGCCCCAAATAATGATTTTATTTTGACTGATAGTGACCTGTTCGTTGCAACAAATTGATGAGCAATG
CTTTTTTATAATGCCAACTTTGTACAAAAAAGCAGGCTCCGCGGCCGCCCCCTTCACCTGTAAGAGACCATTAGGCACCCCAGGCTTTAC
ACTTTATGCTTCCGGCTCGTATAATGTGTGGATTTTGAGTTAGGAGCCGTCGAGATTTTCAGGAGCTAAGGAAGCTAAAATGGAGAAAAA
AATCACTGGATATACCACCGTTGATATATCCCAATGGCATCGTAAAGAACATTTTGAGGCATTTCAGTCAGTTGCTCAATGTACCTATAA
CCAGACCGTTCAGCTGGATATTACGGCCTTTTTAAAGACCGTAAAGAAAAATAAGCACAAGTTTTATCCGGCCTTTATTCACATTCTTGC
CCGCCTGATGAATGCTCATCCGGAGTTCCGTATGGCAATGAAAGACGGTGAGCTGGTGATATGGGATAGTGTTCACCCTTGTTACACCGT
TTTCCATGAGCAAACTGAAACGTTTTCATCGCTCTGGAGTGAATACCACGACGATTTCCGGCAGTTTCTACACATATATTCGCAAGATGT
GGCGTGTTACGGTGAAAACCTGGCCTATTTCCCTAAAGGGTTTATTGAGAATATGTTTTTCGTCTCAGCCAATCCCTGGGTGAGTTTCAC
CAGTTTTGATTTAAACGTGGCCAATATGGACAACTTCTTCGCCCCCGTTTTCACCATGGGCAAATATTATACGCAAGGCGACAAGGTGCT
GATGCCGCTGGCGATTCAGGTTCATCATGCCGTTTGTGATGGCTTCCATGTCGGCAGAATGCTTAATGAATTACAACAGTACTGCGATGA
GTGGCAGGGCGGGGCGTAAACGCGTGGAGCCGGCTTACTAAAAGCCAGATAACAGTATGCGTATTTGCGCGCTGATTTTTGCGGTATAAG
AATATATACTGATATGTATACCCGAAGTATGTCAAAAAGAGGTATGCTATGAAGCAGCGTATTACAGTGACAGTTGACAGCGACAGCTAT
CAGTTGCTCAAGGCATATATGATGTCAATATCTCCGGTCTGGTAAGCACAACCATGCAGAATGAAGCCCGTCGTCTGCGTGCCGAACGCT
GGAAAGCGGAAAATCAGGAAGGGATGGCTGAGGTCGCCCGGTTTATTGAAATGAACGGCTCTTTTGCTGACGAGAACAGGGGCTGGTGAA
ATGCAGTTTAAGGTTTACACCTATAAAAGAGAGAGCCGTTATCGTCTGTTTGTGGATGTACAGAGTGATATTATTGACACGCCCGGCCGA
CGGATGGTGATCCCCCTGGCCAGTGCACGTCTGCTGTCAGATAAAGTCTCCCGTGAACTTTACCCGGTGGTGCATATCGGGGATGAAAGC
TGGCGCATGATGACCACCGATATGGCCAGTGTGCCGGTTTCCGTTATCGGGGAAGAAGTGGCTGATCTCAGCCACCGCGAAAATGACATC
AAAAACGCCATTAACCTGATGTTCTGGGGAATATAAATGTCAGGCTCCCTTATACACAGCCAGTCTGCACCTCGACGGTCTCACATTAAG
GGTGGGCGCGCCGACCCAGCTTTCTTGTACAAAGTTGGCATTATAAGAAAGCATTGCTTATCAATTTGTTGCAACGAACAGGTCACTATC
AGTCAAAATAAAATCATTATTTGCCATCCAGCTGATATCCCCTATAGTGAGTCGTATTACATGGTCATAGCTGTTTCCTGGCAGCTCTGG
CCCGTGTCTCAAAATCTCTGATGTTACATTGCACAAGATAAAAATATATCATCATGAACAATAAAACTGTCTGCTTACATAAACAGTAAT
ACAAGGGGTGTTATGAGCCATATTCAACGGGAAACGTCGAGGCCGCGATTAAATTCCAACATGGATGCTGATTTATATGGGTATAAATGG
GCTCGCGATAATGTCGGGCAATCAGGTGCGACAATCTATCGCTTGTATGGGAAGCCCGATGCGCCAGAGTTGTTTCTGAAACATGGCAAA
GGTAGCGTTGCCAATGATGTTACAGATGAGATGGTCAGACTAAACTGGCTGACGGAATTTATGCCTCTTCCGACCATCAAGCATTTTATC
CGTACTCCTGATGATGCATGGTTACTCACCACTGCGATCCCCGGAAAAACAGCATTCCAGGTATTAGAAGAATATCCTGATTCAGGTGAA
AATATTGTTGATGCGCTGGCAGTGTTCCTGCGCCGGTTGCATTCGATTCCTGTTTGTAATTGTCCTTTTAACAGCGATCGCGTATTTCGT
CTCGCTCAGGCGCAATCACGAATGAATAACGGTTTGGTTGATGCGAGTGATTTTGATGACGAGCGTAATGGCTGGCCTGTTGAACAAGTC
TGGAAAGAAATGCATAAACTTTTGCCATTCTCACCGGATTCAGTCGTCACTCATGGTGATTTCTCACTTGATAACCTTATTTTTGACGAG
GGGAAATTAATAGGTTGTATTGATGTTGGACGAGTCGGAATCGCAGACCGATACCAGGATCTTGCCATCCTATGGAACTGCCTCGGTGAG
TTTTCTCCTTCATTACAGAAACGGCTTTTTCAAAAATATGGTATTGATAATCCTGATATGAATAAATTGCAGTTTCATTTGATGCTCGAT
GAGTTTTTCTAATCAGAATTGGTTAATTGGTTGTAACACTGGCAGAGCATTACGCTGACTTGACGGGACGGCGCAAGCTCATGACCAAAA
TCCCTTAACGTGAGTTACGCGTCGTTCCACTGAGCGTCAGACCCCGTAGAAAAGATCAAAGGATCTTCTTGAGATCCTTTTTTTCTGCGC
GTAATCTGCTGCTTGCAAACAAAAAAACCACCGCTACCAGCGGTGGTTTGTTTGCCGGATCAAGAGCTACCAACTCTTTTTCCGAAGGTA
ACTGGCTTCAGCAGAGCGCAGATACCAAATACTGTCCTTCTAGTGTAGCCGTAGTTAGGCCACCACTTCAAGAACTCTGTAGCACCGCCT
ACATACCTCGCTCTGCTAATCCTGTTACCAGTGGCTGCTGCCAGTGGCGATAAGTCGTGTCTTACCGGGTTGGACTCAAGACGATAGTTA
CCGGATAAGGCGCAGCGGTCGGGCTGAACGGGGGGTTCGTGCACACAGCCCAGCTTGGAGCGAACGACCTACACCGAACTGAGATACCTA
CAGCGTGAGCATTGAGAAAGCGCCACGCTTCCCGAAGGGAGAAAGGCGGACAGGTATCCGGTAAGCGGCAGGGTCGGAACAGGAGAGCGC
ACGAGGGAGCTTCCAGGGGGAAACGCCTGGTATCTTTATAGTCCTGTCGGGTTTCGCCACCTCTGACTTGAGCGTCGATTTTTGTGATGC
TCGTCAGGGGGGCGGAGCCTATGGAAAAACGCCAGCAACGCGGCCTTTTTACGGTTCCTGGCCTTTTGCTGGCCTTTTGCTCACATGTT

M13-F binding site
M13-Reverse binding site
attL1
attL2
Chloramphenicol resistance gene
*ccd*B gene
*Bsa*I site
inverted *Bsa*I site
Kanamycin resistance gene

> *pENTR-AtmiR173aTS-B/c* (4082 bp)

CTTTCCTGCGTTATCCCCTGATTCTGTGGATAACCGTATTACCGCCTTTGAGTGAGCTGATACCGCTCGCCGCAGCCGAACGACCGAGCG
CAGCGAGTCAGTGAGCGAGGAAGCGGAAGAGCGCCCAATACGCAAACCGCCTCTCCCCGCGCGTTGGCCGATTCATTAATGCAGCTGGCA
CGACAGGTTTCCCGACTGGAAAGCGGGCAGTGAGCGCAACGCAATTAATACGCGTACCGCTAGCCAGGAAGAGTTTGTAGAAACGCAAAA
AGGCCATCCGTCAGGATGGCCTTCTGCTTAGTTTGATGCCTGGCAGTTTATGGCGGGCGTCCTGCCCGCCACCCTCCGGGCCGTTGCTTC
ACAACGTTCAAATCCGCTCCCGGCGGATTTGTCCTACTCAGGAGAGCGTTCACCGACAAACAACAGATAAAACGAAAGGCCCAGTCTTCC
GACTGAGCCTTTCGTTTTATTTGATGCCTGGCAGTTCCCTACTCTCGCGTTAACGCTAGCATGGATGTTTTCCCAGTCACGACGTTGTAA
AACGACGGCCAGTCTTAAGCTCGGGCCCCAAATAATGATTTTATTTTGACTGATAGTGACCTGTTCGTTGCAACAAATTGATGAGCAATG
CTTTTTTATAATGCCAACTTTGTACAAAAAAGCAGGCTCCGCGGCCGCCCCCTTCACCTGTA**GTGATTTTTCTCTACAAGCGAATAGACC**
ATTTAAGAGACCATTAGGCACCCCAGGCTTTACACTTTATGCTTCCGGCTCGTATAATGTGTGGATTTTGAGTTAGGAGCCGTCGAGATT
TTCAGGAGCTAAGGAAGCTAAAATGGAGAAAAAAATCACTGGATATACCACCGTTGATATATCCCAATGGCATCGTAAAGAACATTTTGA
GGCATTTCAGTCAGTTGCTCAATGTACCTATAACCAGACCGTTCAGCTGGATATTACGGCCTTTTTAAAGACCGTAAAGAAAAATAAGCA
CAAGTTTTATCCGGCCTTTATTCACATTCTTGCCCGCCTGATGAATGCTCATCCGGAGTTCCGTATGGCAATGAAAGACGGTGAGCTGGT
GATATGGGATAGTGTTCACCCTTGTTACACCGTTTTCCATGAGCAAACTGAAACGTTTTCATCGCTCTGGAGTGAATACCACGACGATTT
CCGGCAGTTTCTACACATATATTCGCAAGATGTGGCGTGTTACGGTGAAAACCTGGCCTATTTCCCTAAAGGGTTTATTGAGAATATGTT
TTTCGTCTCAGCCAATCCCTGGGTGAGTTTCACCAGTTTTGATTTAAACGTGGCCAATATGGACAACTTCTTCGCCCCCGTTTTCACCAT
GGGCAAATATTATACGCAAGGCGACAAGGTGCTGATGCCGCTGGCGATTCAGGTTCATCATGCCGTTTGTGATGGCTTCCATGTCGGCAG
AATGCTTAATGAATTACAACAGTACTGCGATGAGTGGCAGGGCGGGGCGTAAACGCGTGGAGCCGGCTTACTAAAAGCCAGATAACAGTA
TGCGTATTTGCGCGCTGATTTTTGCGGTATAAGAATATATACTGATATGTATACCCGAAGTATGTCAAAAAGAGGTATGCTATGAAGCAG
CGTATTACAGTGACAGTTGACAGCGACAGCTATCAGTTGCTCAAGGCATATATGATGTCAATATCTCCGGTCTGGTAAGCACAACCATGC
AGAATGAAGCCCGTCGTCTGCGTGCCGAACGCTGGAAAGCGGAAAATCAGGAAGGGATGGCTGAGGTCGCCCGGTTTATTGAAATGAACG
GCTCTTTTGCTGACGAGAACAGGGGCTGGTGAAATGCAGTTTAAGGTTTACACCTATAAAAGAGAGAGCCGTTATCGTCTGTTTGTGGAT
GTACAGAGTGATATTATTGACACGCCCGGCCGACGGATGGTGATCCCCCTGGCCAGTGCACGTCTGCTGTCAGATAAAGTCTCCCGTGAA
CTTTACCCGGTGGTGCATATCGGGGATGAAAGCTGGCGCATGATGACCACCGATATGGCCAGTGTGCCGGTTTCCGTTATCGGGGAAGAA
GTGGCTGATCTCAGCCACCGCGAAAATGACATCAAAAACGCCATTAACCTGATGTTCTGGGGAATATAAATGTCAGGCTCCCTTATACAC
AGCCAGTCTGCACCTCGACGGTCTCACATTAAGGGTGGGCGCGCCGACCCAGCTTTCTTGTACAAAGTTGGCATTATAAGAAAGCATTGC
TTATCAATTTGTTGCAACGAACAGGTCACTATCAGTCAAAATAAAATCATTATTTGCCATCCAGCTGATATCCCCTATAGTGAGTCGTAT
TACATGGTCATAGCTGTTTCCTGGCAGCTCTGGCCCGTGTCTCAAAATCTCTGATGTTACATTGCACAAGATAAAAATATATCATCATGA
ACAATAAAACTGTCTGCTTACATAAACAGTAATACAAGGGGTGTTATGAGCCATATTCAACGGGAAACGTCGAGGCCGCGATTAAATTCC
AACATGGATGCTGATTTATATGGGTATAAATGGGCTCGCGATAATGTCGGGCAATCAGGTGCGACAATCTATCGCTTGTATGGGAAGCCC
GATGCGCCAGAGTTGTTTCTGAAACATGGCAAAGGTAGCGTTGCCAATGATGTTACAGATGAGATGGTCAGACTAAACTGGCTGACGGAA
TTTATGCCTCTTCCGACCATCAAGCATTTTATCCGTACTCCTGATGATGCATGGTTACTCACCACTGCGATCCCCGGAAAAACAGCATTC
CAGGTATTAGAAGAATATCCTGATTCAGGTGAAAATATTGTTGATGCGCTGGCAGTGTTCCTGCGCCGGTTGCATTCGATTCCTGTTTGT
AATTGTCCTTTTAACAGCGATCGCGTATTTCGTCTCGCTCAGGCGCAATCACGAATGAATAACGGTTTGGTTGATGCGAGTGATTTTGAT
GACGAGCGTAATGGCTGGCCTGTTGAACAAGTCTGGAAAGAAATGCATAAACTTTTGCCATTCTCACCGGATTCAGTCGTCACTCATGGT
GATTTCTCACTTGATAACCTTATTTTTGACGAGGGGAAATTAATAGGTTGTATTGATGTTGGACGAGTCGGAATCGCAGACCGATACCAG
GATCTTGCCATCCTATGGAACTGCCTCGGTGAGTTTTCTCCTTCATTACAGAAACGGCTTTTTCAAAAATATGGTATTGATAATCCTGAT
ATGAATAAATTGCAGTTTCATTTGATGCTCGATGAGTTTTTCTAATCAGAATTGGTTAATTGGTTGTAACACTGGCAGAGCATTACGCTG
ACTTGACGGGACGGCGCAAGCTCATGACCAAAATCCCTTAACGTGAGTTACGCGTCGTTCCACTGAGCGTCAGACCCCGTAGAAAAGATC
AAAGGATCTTCTTGAGATCCTTTTTTTCTGCGCGTAATCTGCTGCTTGCAAACAAAAAAACCACCGCTACCAGCGGTGGTTTGTTTGCCG
GATCAAGAGCTACCAACTCTTTTTCCGAAGGTAACTGGCTTCAGCAGAGCGCAGATACCAAATACTGTCCTTCTAGTGTAGCCGTAGTTA
GGCCACCACTTCAAGAACTCTGTAGCACCGCCTACATACCTCGCTCTGCTAATCCTGTTACCAGTGGCTGCTGCCAGTGGCGATAAGTCG
TGTCTTACCGGGTTGGACTCAAGACGATAGTTACCGGATAAGGCGCAGCGGTCGGGCTGAACGGGGGGTTCGTGCACACAGCCCAGCTTG
GAGCGAACGACCTACACCGAACTGAGATACCTACAGCGTGAGCATTGAGAAAGCGCCACGCTTCCCGAAGGGAGAAAGGCGGACAGGTAT
CCGGTAAGCGGCAGGGTCGGAACAGGAGAGCGCACGAGGGAGCTTCCAGGGGGAAACGCCTGGTATCTTTATAGTCCTGTCGGGTTTCGC
CACCTCTGACTTGAGCGTCGATTTTTGTGATGCTCGTCAGGGGGGCGGAGCCTATGGAAAAACGCCAGCAACGCGGCCTTTTTACGGTTC
CTGGCCTTTTGCTGGCCTTTTGCTCACATGTT

AtmiR173a target site
AtTAS1c-derived spacer
M13-F binding site
M13-Reverse binding site
attL1
attL2
Chloramphenicol resistance gene
*ccd*B gene
*Bsa*I site
inverted *Bsa*I site
Kanamycin resistance gene

>pENTR-NbmiR482aTS-B/c (4082 bp)

CTTTCCTGCGTTATCCCCTGATTCTGTGGATAACCGTATTACCGCCTTTGAGTGAGCTGATACCGCTCGCCGCAGCCGAACGACCGAGCG
CAGCGAGTCAGTGAGCGAGGAAGCGGAAGAGCGCCCAATACGCAAACCGCCTCTCCCCGCGCGTTGGCCGATTCATTAATGCAGCTGGCA
CGACAGGTTTCCCGACTGGAAAGCGGGCAGTGAGCGCAACGCAATTAATACGCGTACCGCTAGCCAGGAAGAGTTTGTAGAAACGCAAAA
AGGCCATCCGTCAGGATGGCCTTCTGCTTAGTTTGATGCCTGGCAGTTTATGGCGGGCGTCCTGCCCGCCACCCTCCGGGCCGTTGCTTC
ACAACGTTCAAATCCGCTCCCGGCGGATTTGTCCTACTCAGGAGAGCGTTCACCGACAAACAACAGATAAAACGAAAGGCCCAGTCTTCC
GACTGAGCCTTTCGTTTTATTTGATGCCTGGCAGTTCCCTACTCTCGCGTTAACGCTAGCATGGATGTTTTCCCAGTCACGACGTTGTAA
AACGACGGCCAGTCTTAAGCTCGGGCCCCAAATAATGATTTTATTTTGACTGATAGTGACCTGTTCGTTGCAACAAATTGATGAGCAATG
CTTTTTTATAATGCCAACTTTGTACAAAAAAGCAGGCTCCGCGGCCGCCCCCTTCACCTGTA**GTGGTATGGGGGGAGTCGGGAA****TAGACC**
ATTTAAGAGACCATTAGGCACCCCAGGCTTTACACTTTATGCTTCCGGCTCGTATAATGTGTGGATTTTGAGTTAGGAGCCGTCGAGATT
TTCAGGAGCTAAGGAAGCTAAAATGGAGAAAAAAATCACTGGATATACCACCGTTGATATATCCCAATGGCATCGTAAAGAACATTTTGA
GGCATTTCAGTCAGTTGCTCAATGTACCTATAACCAGACCGTTCAGCTGGATATTACGGCCTTTTTAAAGACCGTAAAGAAAAATAAGCA
CAAGTTTTATCCGGCCTTTATTCACATTCTTGCCCGCCTGATGAATGCTCATCCGGAGTTCCGTATGGCAATGAAAGACGGTGAGCTGGT
GATATGGGATAGTGTTCACCCTTGTTACACCGTTTTCCATGAGCAAACTGAAACGTTTTCATCGCTCTGGAGTGAATACCACGACGATTT
CCGGCAGTTTCTACACATATATTCGCAAGATGTGGCGTGTTACGGTGAAAACCTGGCCTATTTCCCTAAAGGGTTTATTGAGAATATGTT
TTTCGTCTCAGCCAATCCCTGGGTGAGTTTCACCAGTTTTGATTTAAACGTGGCCAATATGGACAACTTCTTCGCCCCCGTTTTCACCAT
GGGCAAATATTATACGCAAGGCGACAAGGTGCTGATGCCGCTGGCGATTCAGGTTCATCATGCCGTTTGTGATGGCTTCCATGTCGGCAG
AATGCTTAATGAATTACAACAGTACTGCGATGAGTGGCAGGGCGGGGCGTAAACGCGTGGAGCCGGCTTACTAAAAGCCAGATAACAGTA
TGCGTATTTGCGCGCTGATTTTTGCGGTATAAGAATATATACTGATATGTATACCCGAAGTATGTCAAAAAGAGGTATGCTATGAAGCAG
CGTATTACAGTGACAGTTGACAGCGACAGCTATCAGTTGCTCAAGGCATATATGATGTCAATATCTCCGGTCTGGTAAGCACAACCATGC
AGAATGAAGCCCGTCGTCTGCGTGCCGAACGCTGGAAAGCGGAAAATCAGGAAGGGATGGCTGAGGTCGCCCGGTTTATTGAAATGAACG
GCTCTTTTGCTGACGAGAACAGGGGCTGGTGAAATGCAGTTTAAGGTTTACACCTATAAAAGAGAGAGCCGTTATCGTCTGTTTGTGGAT
GTACAGAGTGATATTATTGACACGCCCGGCCGACGGATGGTGATCCCCCTGGCCAGTGCACGTCTGCTGTCAGATAAAGTCTCCCGTGAA
CTTTACCCGGTGGTGCATATCGGGGATGAAAGCTGGCGCATGATGACCACCGATATGGCCAGTGTGCCGGTTTCCGTTATCGGGGAAGAA
GTGGCTGATCTCAGCCACCGCGAAAATGACATCAAAAACGCCATTAACCTGATGTTCTGGGGAATATAAATGTCAGGCTCCCTTATACAC
AGCCAGTCTGCACCTCGACGGTCTCACATTAAGGGTGGGCGCGCCGACCCAGCTTTCTTGTACAAAGTTGGCATTATAAGAAAGCATTGC
TTATCAATTTGTTGCAACGAACAGGTCACTATCAGTCAAAATAAAATCATTATTTGCCATCCAGCTGATATCCCCTATAGTGAGTCGTAT
TACATGGTCATAGCTGTTTCCTGGCAGCTCTGGCCCGTGTCTCAAAATCTCTGATGTTACATTGCACAAGATAAAAATATATCATCATGA
ACAATAAAACTGTCTGCTTACATAAACAGTAATACAAGGGGTGTTATGAGCCATATTCAACGGGAAACGTCGAGGCCGCGATTAAATTCC
AACATGGATGCTGATTTATATGGGTATAAATGGGCTCGCGATAATGTCGGGCAATCAGGTGCGACAATCTATCGCTTGTATGGGAAGCCC
GATGCGCCAGAGTTGTTTCTGAAACATGGCAAAGGTAGCGTTGCCAATGATGTTACAGATGAGATGGTCAGACTAAACTGGCTGACGGAA
TTTATGCCTCTTCCGACCATCAAGCATTTTATCCGTACTCCTGATGATGCATGGTTACTCACCACTGCGATCCCCGGAAAAACAGCATTC
CAGGTATTAGAAGAATATCCTGATTCAGGTGAAAATATTGTTGATGCGCTGGCAGTGTTCCTGCGCCGGTTGCATTCGATTCCTGTTTGT
AATTGTCCTTTTAACAGCGATCGCGTATTTCGTCTCGCTCAGGCGCAATCACGAATGAATAACGGTTTGGTTGATGCGAGTGATTTTGAT
GACGAGCGTAATGGCTGGCCTGTTGAACAAGTCTGGAAAGAAATGCATAAACTTTTGCCATTCTCACCGGATTCAGTCGTCACTCATGGT
GATTTCTCACTTGATAACCTTATTTTTGACGAGGGGAAATTAATAGGTTGTATTGATGTTGGACGAGTCGGAATCGCAGACCGATACCAG
GATCTTGCCATCCTATGGAACTGCCTCGGTGAGTTTTCTCCTTCATTACAGAAACGGCTTTTTCAAAAATATGGTATTGATAATCCTGAT
ATGAATAAATTGCAGTTTCATTTGATGCTCGATGAGTTTTTCTAATCAGAATTGGTTAATTGGTTGTAACACTGGCAGAGCATTACGCTG
ACTTGACGGGACGGCGCAAGCTCATGACCAAAATCCCTTAACGTGAGTTACGCGTCGTTCCACTGAGCGTCAGACCCCGTAGAAAAGATC
AAAGGATCTTCTTGAGATCCTTTTTTTCTGCGCGTAATCTGCTGCTTGCAAACAAAAAAACCACCGCTACCAGCGGTGGTTTGTTTGCCG
GATCAAGAGCTACCAACTCTTTTTCCGAAGGTAACTGGCTTCAGCAGAGCGCAGATACCAAATACTGTCCTTCTAGTGTAGCCGTAGTTA
GGCCACCACTTCAAGAACTCTGTAGCACCGCCTACATACCTCGCTCTGCTAATCCTGTTACCAGTGGCTGCTGCCAGTGGCGATAAGTCG
TGTCTTACCGGGTTGGACTCAAGACGATAGTTACCGGATAAGGCGCAGCGGTCGGGCTGAACGGGGGGTTCGTGCACACAGCCCAGCTTG
GAGCGAACGACCTACACCGAACTGAGATACCTACAGCGTGAGCATTGAGAAAGCGCCACGCTTCCCGAAGGGAGAAAGGCGGACAGGTAT
CCGGTAAGCGGCAGGGTCGGAACAGGAGAGCGCACGAGGGAGCTTCCAGGGGGAAACGCCTGGTATCTTTATAGTCCTGTCGGGTTTCGC
CACCTCTGACTTGAGCGTCGATTTTTGTGATGCTCGTCAGGGGGGCGGAGCCTATGGAAAAACGCCAGCAACGCGGCCTTTTTACGGTTC
CTGGCCTTTTGCTGGCCTTTTGCTCACATGTT

NbmiR482a target site
AtTAS1c-derived spacer
M13-F binding site
M13-Reverse binding site
attL1
attL2
Chloramphenicol resistance gene
*ccd*B gene
*Bsa*I site
inverted *Bsa*I site
Kanamycin resistance gene

>*pMDC32B-B/c* (11602 bp)

CCAGCCAGCCAACAGCTCCCCGACCGGCAGCTCGGCACAAAATCACCACTCGATACAGGCAGCCCATCAGTCCGGGACGGCGTCAGCGGG
AGAGCCGTTGTAAGGCGGCAGACTTTGCTCATGTTACCGATGCTATTCGGAAGAACGGCAACTAAGCTGCCGGGTTTGAAACACGGATGA
TCTCGCGGAGGGTAGCATGTTGATTGTAACGATGACAGAGCGTTGCTGCCTGTGATCACCGCGGTTTCAAAATCGGCTCCGTCGATACTA
TGTTATACGCCAACTTTGAAAACAACTTTGAAAAAGCTGTTTTCTGGTATTTAAGGTTTTAGAATGCAAGGAACAGTGAATTGGAGTTCG
TCTTGTTATAATTAGCTTCTTGGGGTATCTTTAAATACTGTAGAAAAGAGGAAGGAAATAATAAATGGCTAAAATGAGAATATCACCGGA
ATTGAAAAAACTGATCGAAAAATACCGCTGCGTAAAAGATACGGAAGGAATGTCTCCTGCTAAGGTATATAAGCTGGTGGGAGAAAATGA
AAACCTATATTTAAAAATGACGGACAGCCGGTATAAAGGGACCACCTATGATGTGGAACGGGAAAAGGACATGATGCTATGGCTGGAAGG
AAAGCTGCCTGTTCCAAAGGTCCTGCACTTTGAACGGCATGATGGCTGGAGCAATCTGCTCATGAGTGAGGCCGATGGCGTCCTTTGCTC
GGAAGAGTATGAAGATGAACAAAGCCCTGAAAAGATTATCGAGCTGTATGCGGAGTGCATCAGGCTCTTTCACTCCATCGACATATCGGA
TTGTCCCTATACGAATAGCTTAGACAGCCGCTTAGCCGAATTGGATTACTTACTGAATAACGATCTGGCCGATGTGGATTGCGAAAACTG
GGAAGAAGACACTCCATTTAAAGATCCGCGCGAGCTGTATGATTTTTTAAAGACGGAAAAGCCCGAAGAGGAACTTGTCTTTTCCCACGG
CGACCTGGGAGACAGCAACATCTTTGTGAAAGATGGCAAAGTAAGTGGCTTTATTGATCTTGGGAGAAGCGGCAGGGCGGACAAGTGGTA
TGACATTGCCTTCTGCGTCCGGTCGATCAGGGAGGATATCGGGGAAGAACAGTATGTCGAGCTATTTTTTGACTTACTGGGGATCAAGCC
TGATTGGGAGAAAATAAAATATTATATTTTACTGGATGAATTGTTTTAGTACCTAGAATGCATGACCAAAATCCCTTAACGTGAGTTTTC
GTTCCACTGAGCGTCAGACCCCGTAGAAAAGATCAAAGGATCTTCTTGAGATCCTTTTTTTCTGCGCGTAATCTGCTGCTTGCAAACAAA
AAAACCACCGCTACCAGCGGTGGTTTGTTTGCCGGATCAAGAGCTACCAACTCTTTTTCCGAAGGTAACTGGCTTCAGCAGAGCGCAGAT
ACCAAATACTGTCCTTCTAGTGTAGCCGTAGTTAGGCCACCACTTCAAGAACTCTGTAGCACCGCCTACATACCTCGCTCTGCTAATCCT
GTTACCAGTGGCTGCTGCCAGTGGCGATAAGTCGTGTCTTACCGGGTTGGACTCAAGACGATAGTTACCGGATAAGGCGCAGCGGTCGGG
CTGAACGGGGGGTTCGTGCACACAGCCCAGCTTGGAGCGAACGACCTACACCGAACTGAGATACCTACAGCGTGAGCTATGAGAAAGCGC
CACGCTTCCCGAAGGGAGAAAGGCGGACAGGTATCCGGTAAGCGGCAGGGTCGGAACAGGAGAGCGCACGAGGGAGCTTCCA GGGGGAAA
CGCCTGGTATCTTTATAGTCCTGTCGGGTTTCGCCACCTCTGACTTGAGCGTCGATTTTTGTGATGCTCGTCAGGGGGGCGGAGCCTATG
GAAAAACGCCAGCAACGCGGCCTTTTTACGGTTCCTGGCCTTTTGCTGGCCTTTTGCTCACATGTTCTTTCCTGCGTTATCCCCTGATTC
TGTGGATAACCGTATTACCGCCTTTGAGTGAGCTGATACCGCTCGCCGCAGCCGAACGACCGAGCGCAGCGAGTCAGTGAGCGAGGAAGC
GGAAGAGCGCCTGATGCGGTATTTTCTCCTTACGCATCTGTGCGGTATTTCACACCGCATATGGTGCACTCTCAGTACAATCTGCTCTGA
TGCCGCATAGTTAAGCCAGTATACACTCCGCTATCGCTACGTGACTGGGTCATGGCTGCGCCCCGACACCCGCCAACACCCGCTGACGCG
CCCTGACGGGCTTGTCTGCTCCCGGCATCCGCTTACAGACAAGCTGTGACCGTCTCCGGGAGCTGCATGTGTCAGAGGTTTTCACCGTCA
TCACCGAAACGCGCGAGGCAGGGTGCCTTGATGTGGGCGCCGGCGGTCGAGTGGCGACGGCGCGGCTTGTCCGCGCCCTGGTAGATTGCC
TGGCCGTAGGCCAGCCATTTTTGAGCGGCCAGCGGCCGCGATAGGCCGACGCGAAGCGGCGGGGCGTAGGGAGCGCAGCGACCGAAGGGT
AGGCGCTTTTTGCAGCTCTTCGGCTGTGCGCTGGCCAGACAGTTATGCACAGGCCAGGCGGGTTTTAAGAGTTTTAATAAGTTTTAAAGA
GTTTTAGGCGGAAAAATCGCCTTTTTTCTCTTTTATATCAGTCACTTACATGTGTGACCGGTTCCCAATGTACGGCTTTGGGTTCCCAAT
GTACGGGTTCCGGTTCCCAATGTACGGCTTTGGGTTCCCAATGTACGTGCTATCCACAGGAAAGAGAACTTTTCGACCTTTTTCCCCTGC
TAGGGCAATTTGCCCTAGCATCTGCTCCGTACATTAGGAACCGGCGGATGCTTCGCCCTCGATCAGGTTGCGGTAGCGCATGACTAGGAT
CGGGCCAGCCTGCCCCGCCTCCTCCTTCAAATCGTACTCCGGCAGGTCATTTGACCCGATCAGCTTGCGCACGGTGAAACAGAACTTCTT
GAACTCTCCGGCGCTGCCACTGCGTTCGTAGATCGTCTTGAACAACCATCTGGCTTCTGCCTTGCCTGCGGCGCGGCGTGCCAGGCGGTA
GAGAAAACGGCCGATGCCGGGATCGATCAAAAAGTAATCGGGGTGAACCGTCAGCACGTCCGGGTTCTTGCCTTCTGTGATCTCGCGGTA
CATCCAATCAGCTAGCTCGATCTCGATGTACTCCGGCCGCCCGGTTTCGCTCTTTACGATCTTGTAGCGGCTAATCAAGGCTTCACCCTC
GGATACCGTCACCAGGCGGCCGTTCTTGGCCTTCTTCGTACGCTGCATGGCAACGTGCGTGGTGTTTAACCGAATGCAGGTTTCTACCAG
GTCGTCTTTCTGCTTTCCGCCATCGGCTCGCCGGCAGAACTTGAGTACGTCCGCAACGTGTGGACGGAACACGCGGCCGGGCTTGTCTCC
CTTCCCTTCCCGGTATCGGTTCATGGATTCGGTTAGATGGGAAACCGCCATCAGTACCAGGTCGTAATCCCACACACTGGCCATGCCGGC
CGGCCCTGCGGAAACCTCTACGTGCCCGTCTGGAAGCTCGTAGCGGATCACCTCGCCAGCTCGTCGGTCACGCTTCGACAGACGGAAAAC
GGCCACGTCCATGATGCTGCGACTATCGCGGGTGCCCACGTCATAGAGCATCGGAACGAAAAAATCTGGTTGCTCGTCGCCCTTGGGCGG
CTTCCTAATCGACGGCGCACCGGCTGCCGGCGGTTGCCGGGATTCTTTGCGGATTCGATCAGCGGCCGCTTGCCACGATTCACCGGGGCG
TGCTTCTGCCTCGATGCGTTGCCGCTGGGCGGCCTGCGCGGCCTTCAACTTCTCCACCAGGTCATCACCCAGCGCCGCGCCGATTTGTAC
CGGGCCGGATGGTTTGCGACCGTCACGCCGATTCCTCGGGCTTGGGGGTTCCAGTGCCATTGCAGGGCCGGCAGACAACCCAGCCGCTTA
CGCCTGGCCAACCGCCCGTTCCTCCACACATGGGGCATTCCACGGCGTCGGTGCCTGGTTGTTCTTGATTTTCCATGCCGCCTCCTTTAG
CCGCTAAAATTCATCTACTCATTTATTCATTTGCTCATTTACTCTGGTAGCTGCGCGATGTATTCAGATAGCAGCTCGGTAATGGTCTTG
CCTTGGCGTACCGCGTACATCTTCAGCTTGGTGTGATCCTCCGCCGGCAACTGAAAGTTGACCCGCTTCATGGCTGGCGTGTCTGCCAGG
CTGGCCAACGTTGCAGCCTTGCTGCTGCGTGCGCTCGGACGGCCGGCACTTAGCGTGTTTGTGCTTTTGCTCATTTTCTCTTTACCTCAT
TAACTCAAATGAGTTTTGATTTAATTTCAGCGGCCAGCGCCTGGACCTCGCGGGCAGCGTCGCCCTCGGGTTCTGATTCAAGAACGGTTG
TGCCGGCGGCGGCAGTGCCTGGGTAGCTCACGCGCTGCGTGATACGGGACTCAAGAATGGGCAGCTCGTACCCGGCCAGCGCCTCGGCAA
CCTCACCGCCGATGCGCGTGCCTTTGATCGCCCGCGACACGACAAAGGCCGCTTGTAGCCTTCCATCCGTGACCTCAATGCGCTGCTTAA
CCAGCTCCACCAGGTCGGCGGTGGCCCATATGTCGTAAGGGCTTGGCTGCACCGGAATCAGCACGAAGTCGGCTGCCTTGATCGCGGACA
CAGCCAAGTCCGCCGCCTGGGGCGCTCCGTCGATCACTACGAAGTCGCGCCGGCCGATGGCCTTCACGTCGCGGTCAATCGTCGGGCGGT
CGATGCCGACAACGGTTAGCGGTTGATCTTCCCGCACGGCCGCCCAATCGCGGGCACTGCCCTGGGGATCGGAATCGACTAACAGAACAT
CGGCCCCGGCGAGTTGCAGGGCGCGGGCTAGATGGGTTGCGATGGTCGTCTTGCCTGACCCGCCTTTCTGGTTAAGTACAGCGATAACCT
TCATGCGTTCCCCTTGCGTATTTGTTTATTTACTCATCGCATCATATACGCAGCGACCGCATGACGCAAGCTGTTTTACTCAAATACACA
TCACCTTTTTAGACGGCGGCGCTCGGTTTCTTCAGCGGCCAAGCTGGCCGGCCAGGCCGCCAGCTTGGCATCAGACAAAC CGGCCAGGAT
TTCATGCAGCCGCACGGTTGAGACGTGCGCGGGCGGCTCGAACACGTACCCGGCCGCGATCATCTCCGCCTCGATCTCTTCGGTAATGAA
AAACGGTTCGTCCTGGCCGTCCTGGTGCGGTTTCATGCTTGTTCCTCTTGGCGTTCATTCTCGGCGGCCGCCAGGGCGTCGGCCTCGGTC
AATGCGTCCTCACGGAAGGCACCGCGCCGCCTGGCCTCGGTGGGCGTCACTTCCTCGCTGCGCTCAAGTGCGCGGTACAGGGTCGAGCGA
TGCACGCCAAGCAGTGCAGCCGCCTCTTTCACGGTGCGGCCTTCCTGGTCGATCAGCTCGCGGGCGTGCGCGATCTGTGCCGGGGTGAGG
GTAGGGCGGGGGCCAAACTTCACGCCTCGGGCCTTGGCGGCCTCGCGCCCGCTCCGGGTGCGGTCGATGATTAGGGAACGCTCGAACTCG

GCAATGCCGGCGAACACGGTCAACACCATGCGGCCGGCCGGCGTGGTGGTGTCGGCCCACGGCTCTGCCAGGCTACGCAGGCCCGCGCCG
GCCTCCTGGATGCGCTCGGCAATGTCCAGTAGGTCGCGGGTGCTGCGGGCCAGGCGGTCTAGCCTGGTCACTGTCACAACGTCGCCAGGG
CGTAGGTGGTCAAGCATCCTGGCCAGCTCCGGGCGGTCGCGCCTGGTGCCGGTGATCTTCTCGGAAAACAGCTTGGTGCAGCCGGCCGCG
TGCAGTTCGGCCCGTTGGTTGGTCAAGTCCTGGTCGTCGGTGCTGACGCGGGCATAGCCCAGCAGGCCAGCGGCGGCGCTCTTGTTCATG
GCGTAATGTCTCCGGTTCTAGTCGCAAGTATTCTACTTTATGCGACTAAAACACGCGACAAGAAAACGCCAGGAAAAGGGCAGGGCGGCA
GCCTGTCGCGTAACTTAGGACTTGTGCGACATGTCGTTTTCAGAAGACGGCTGCACTGAACGTCAGAAGCCGACTGCACTATAGCAGCGG
AGGGGTTGGATCAAAGTACTTTGATCCCGAGGGGAACCCTGTGGTTGGCATGCACATACAAATGGACGAACGGATAAACCTTTTCACGCC
CTTTTAAATATCCGTTATTCTAATAAACGCTCTTTTCTCTTAGGTTTACCCGCCAATATATCCTGTCAAACACTGATAGTTTAAACTGAA
GGCGGGAAACGACAATCTGATCCAAGCTCAAGCTGCTCTAGCATTCGCCATTCAGGCTGCGCAACTGTTGGGAAGGGCGATCGGTGCGGG
CCTCTTCGCTATTACGCCAGCTGGCGAAAGGGGGATGTGCTGCAAGGCGATTAAGTTGGGTAACGCCAGGGTTTTCCCAGTCACGACGTT
GTAAAACGACGGCCAGTGCCAAGCTTGGCGTGCCTGCAGGTCAACATGGTGGAGCACGACACACTTGTCTACTCCAAAAATATCAAAGAT
ACAGTCTCAGAAGACCAAAGGGCAATTGAGACTTTTCAACAAAGGGTAATATCCGGAAACCTCCTCGGATTCCATTGCCCAGCTATCTGT
CACTTTATTGTGAAGATAGTGGAAAAGGAAGGTGGCTCCTACAAATGCCATCATTGCGATAAAGGAAAGGCCATCGTTGAAGATGCCTCT
GCCGACAGTGGTCCCAAAGATGGACCCCCACCCACGAGGAGCATCGTGGAAAAAGAAGACGTTCCAACCACGTCTTCAAAGCAAGTGGAT
TGATGTGATAACATGGTGGAGCACGACACACTTGTCTACTCCAAAAATATCAAAGATACAGTCTCAGAAGACCAAAGGGCAATTGAGACT
TTTCAACAAAGGGTAATATCCGGAAACCTCCTCGGATTCCATTGCCCAGCTATCTGTCACTTTATTGTGAAGATAGTGGAAAAGGAAGGT
GGCTCCTACAAATGCCATCATTGCGATAAAGGAAAGGCCATCGTTGAAGATGCCTCTGCCGACAGTGGTCCCAAAGATGGACCCCCACCC
ACGAGGAGCATCGTGGAAAAAGAAGACGTTCCAACCACGTCTTCAAAGCAAGTGGATTGATGTGATATCTCCACTGACGTAAGGGATGAC
GCACAATCCCACTATCCTTCGCAAGACCCTTCCTCTATATAAGGAAGTTCATTTCATTTGGAGAGGACCTCGACTCTAGAGGATCCCCGG
GTACCGGGCCCCCCCTCGAGGCGCGCCAAGCTATCAAACAAGTTTGTACAAAAAAGCAGGCTCCGCGGCCGCCCCCTTCACCTGTAAGAG
ACCATTAGGCACCCCAGGCTTTACACTTTATGCTTCCGGCTCGTATAATGTGTGGATTTTGAGTTAGGAGCCGTCGAGATTTTCAGGAGC
TAAGGAAGCTAAAATGGAGAAAAAAATCACTGGATATACCACCGTTGATATATCCCAATGGCATCGTAAAGAACATTTTGAGGCATTTCA
GTCAGTTGCTCAATGTACCTATAACCAGACCGTTCAGCTGGATATTACGGCCTTTTTAAAGACCGTAAAGAAAAATAAGCACAAGTTTTA
TCCGGCCTTTATTCACATTCTTGCCCGCCTGATGAATGCTCATCCGGAGTTCCGTATGGCAATGAAAGACGGTGAGCTGGTGATATGGGA
TAGTGTTCACCCTTGTTACACCGTTTTCCATGAGCAAACTGAAACGTTTTCATCGCTCTGGAGTGAATACCACGACGATTTCCGGCAGTT
TCTACACATATATTCGCAAGATGTGGCGTGTTACGGTGAAAACCTGGCCTATTTCCCTAAAGGGTTTATTGAGAATATGTTTTTCGTCTC
AGCCAATCCCTGGGTGAGTTTCACCAGTTTTGATTTAAACGTGGCCAATATGGACAACTTCTTCGCCCCCGTTTTCACCATGGGCAAATA
TTATACGCAAGGCGACAAGGTGCTGATGCCGCTGGCGATTCAGGTTCATCATGCCGTTTGTGATGGCTTCCATGTCGGCAGAATGCTTAA
TGAATTACAACAGTACTGCGATGAGTGGCAGGGCGGGGCGTAAACGCGTGGAGCCGGCTTACTAAAAGCCAGATAACAGTATGCGTATTT
GCGCGCTGATTTTTGCGGTATAAGAATATATACTGATATGTATACCCGAAGTATGTCAAAAAGAGGTATGCTATGAAGCAGCGTATTACA
GTGACAGTTGACAGCGACAGCTATCAGTTGCTCAAGGCATATATGATGTCAATATCTCCGGTCTGGTAAGCACAACCATGCAGAATGAAG
CCCGTCGTCTGCGTGCCGAACGCTGGAAAGCGGAAAATCAGGAAGGGATGGCTGAGGTCGCCCGGTTTATTGAAATGAACGGCTCTTTTG
CTGACGAGAACAGGGGCTGGTGAAATGCAGTTTAAGGTTTACACCTATAAAAGAGAGAGCCGTTATCGTCTGTTTGTGGATGTACAGAGT
GATATTATTGACACGCCCGGCCGACGGATGGTGATCCCCCTGGCCAGTGCACGTCTGCTGTCAGATAAAGTCTCCCGTGAACTTTACCCG
GTGGTGCATATCGGGGATGAAAGCTGGCGCATGATGACCACCGATATGGCCAGTGTGCCGGTTTCCGTTATCGGGGAAGAAGTGGCTGAT
CTCAGCCACCGCGAAAATGACATCAAAAACGCCATTAACCTGATGTTCTGGGGAATATAAATGTCAGGCTCCCTTATACACAGCCAGTCT
GCACCTCGACGGTCTCACATTAAGGGTGGGCGCGCCGACCCAGCTTTCTTGTACAAAGTGGTTCGATAATTCCTTAATTAACTAGTTCTA
GAGCGGCCGCCCACCGCGGTGGAGCTCGAATTTCCCCGATCGTTCAAACATTTGGCAATAAAGTTTCTTAAGATTGAATCCTGTTGCCGG
TCTTGCGATGATTATCATATAATTTCTGTTGAATTACGTTAAGCATGTAATAATTAACATGTAATGCATGACGTTATTTATGAGATGGGT
TTTTATGATTAGAGTCCCGCAATTATACATTTAATACGCGATAGAAAACAAAATATAGCGCGCAAACTAGGATAAATTATCGCGCGCGGT
GTCATCTATGTTACTGAATTCGTAATCATGGTCATAGCTGTTTCCTGTGTGAAATTGTTATCCGCTCACAATTCCACACAACATACGAGC
CGGAAGCATAAAGTGTAAAGCCTGGGGTGCCTAATGAGTGAGCTAACTCACATTAATTGCGTTGCGCTCACTGCCCGCTTTCCAGTCGGG
AAACCTGTCGTGCCAGCTGCATTAATGAATCGGCCAACGCGCGGGGAGAGGCGGTTTGCGTATTGGCTAGAGCAGCTTGCCAACATGGTG
GAGCACGACACTCTCGTCTACTCCAAGAATATCAAAGATACAGTCTCAGAAGACCAAAGGGCTATTGAGACTTTTCAACAAAGGGTAATA
TCGGGAAACCTCCTCGGATTCCATTGCCCAGCTATCTGTCACTTCATCAAAAGGACAGTAGAAAAGGAAGGTGGCACCTACAAATGCCAT
CATTGCGATAAAGGAAAGGCTATCGTTCAAGATGCCTCTGCCGACAGTGGTCCCAAAGATGGACCCCCACCCACGAGGAGCATCGTGGAA
AAAGAAGACGTTCCAACCACGTCTTCAAAGCAAGTGGATTGATGTGATAACATGGTGGAGCACGACACTCTCGTCTACTCCAAGAATATC
AAAGATACAGTCTCAGAAGACCAAAGGGCTATTGAGACTTTTCAACAAAGGGTAATATCGGGAAACCTCCTCGGATTCCATTGCCCAGCT
ATCTGTCACTTCATCAAAAGGACAGTAGAAAAGGAAGGTGGCACCTACAAATGCCATCATTGCGATAAAGGAAAGGCTATCGTTCAAGAT
GCCTCTGCCGACAGTGGTCCCAAAGATGGACCCCCACCCACGAGGAGCATCGTGGAAAAAGAAGACGTTCCAACCACGTCTTCAAAGCAA
GTGGATTGATGTGATATCTCCACTGACGTAAGGGATGACGCACAATCCCACTATCCTTCGCAAGACCTTCCTCTATATAAGGAAGTTCAT
TTCATTTGGAGAGGACACGCTGAAATCACCAGTCTCTCTCTACAAATCTATCTCTCTCGAGCTTTCGCAGATCCCGGGGGGCAATGAGAT
ATGAAAAAGCCTGAACTCACCGCGACGTCTGTCGAGAAGTTTCTGATCGAAAAGTTCGACAGCGTCTCCGACCTGATGCAGCTCTCGGAG
GGCGAAGAATCTCGTGCTTTCAGCTTCGATGTAGGAGGGCGTGGATATGTCCTGCGGGTAAATAGCTGCGCCGATGGTTTCTACAAAGAT
CGTTATGTTTATCGGCACTTTGCATCGGCCGCGCTCCCGATTCCGGAAGTGCTTGACATTGGGGAGTTTAGCGAGAGCCTGACCTATTGC
ATCTCCCGCCGTGCACAGGGTGTCACGTTGCAAGACCTGCCTGAAACCGAACTGCCCGCTGTTCTACAACCGGTCGCGGAGGCTATGGAT
GCGATCGCTGCGGCCGATCTTAGCCAGACGAGCGGGTTCGGCCCATTCGGACCGCAAGGAATCGGTCAATACACTACATGGCGTGATTTC
ATATGCGCGATTGCTGATCCCCATGTGTATCACTGGCAAACTGTGATGGACGACACCGTCAGTGCGTCCGTCGCGCAGGCTCTCGATGAG
CTGATGCTTTGGGCCGAGGACTGCCCCGAAGTCCGGCACCTCGTGCACGCGGATTTCGGCTCCAACAATGTCCTGACGGACAATGGCCGC
ATAACAGCGGTCATTGACTGGAGCGAGGCGATGTTCGGGGATTCCCAATACGAGGTCGCCAACATCTTCTTCTGGAGGCCGTGGTTGGCT
TGTATGGAGCAGCAGACGCGCTACTTCGAGCGGAGGCATCCGGAGCTTGCAGGATCGCCACGACTCCGGGCGTATATGCTCCGCATTGGT
CTTGACCAACTCTATCAGAGCTTGGTTGACGGCAATTTCGATGATGCAGCTTGGGCGCAGGGTCGATGCGACGCAATCGTCCGATCCGGA
GCCGGGACTGTCGGGCGTACACAAATCGCCCGCAGAAGCGCGGCCGTCTGGACCGATGGCTGTGTAGAAGTACTCGCCGATAGTGGAAAC

CGACGCCCCAGCACTCGTCCGAGGGCAAAGAAATAGAGTAGATGCCGACCGGATCTGTCGATCGACAAGCTCGAGTTTCTCCATAATAAT
GTGTGAGTAGTTCCCAGATAAGGGAATTAGGGTTCCTATAGGGTTTCGCTCATGTGTTGAGCATATAAGAAACCCTTAGTATGTATTTGT
ATTTGTAAAATACTTCTATCAATAAAATTTCTAATTCCTAAAACCAAAATCCAGTACTAAAATCCAGATCCCCCGAATTAATTCGGCGTT
AATTCAGTACATTAAAAACGTCCGCAATGTGTTATTAAGTTGTCTAAGCGTCAATTTGTTTACACCACAATATATCCTGCCA

T-DNA right border
T-DNA left border
*ccd*B gene
*Bsa*I site
Inverted *Bsa*I site
Chloramphenicol resistance gene
attB1
attB2
Nos terminator
CaMV promoter
kanamycin resistance gene
Hygromycin resistance gene
2x35S CaMV promoter
CaMV terminator

> *pMDC32B-AtmiR173aTS-B/c* (11635 bp)

CCAGCCAGCCAACAGCTCCCCGACCGGCAGCTCGGCACAAAATCACCACTCGATACAGGCAGCCCATCAGTCCGGGACGGCGTCAGCGGG
AGAGCCGTTGTAAGGCGGCAGACTTTGCTCATGTTACCGATGCTATTCGGAAGAACGGCAACTAAGCTGCCGGGTTTGAAACACGGATGA
TCTCGCGGAGGGTAGCATGTTGATTGTAACGATGACAGAGCGTTGCTGCCTGTGATCACCGCGGTTTCAAAATCGGCTCCGTCGATACTA
TGTTATACGCCAACTTTGAAAACAACTTTGAAAAAGCTGTTTTCTGGTATTTAAGGTTTTAGAATGCAAGGAACAGTGAATTGGAGTTCG
TCTTGTTATAATTAGCTTCTTGGGGTATCTTTAAATACTGTAGAAAAGAGGAAGGAAATAATAAATGGCTAAAATGAGAATATCACCGGA
ATTGAAAAAACTGATCGAAAAATACCGCTGCGTAAAAGATACGGAAGGAATGTCTCCTGCTAAGGTATATAAGCTGGTGGGAGAAAATGA
AAACCTATATTTAAAAATGACGGACAGCCGGTATAAAGGGACCACCTATGATGTGGAACGGGAAAAGGACATGATGCTATGGCTGGAAGG
AAAGCTGCCTGTTCCAAAGGTCCTGCACTTTGAACGGCATGATGGCTGGAGCAATCTGCTCATGAGTGAGGCCGATGGCGTCCTTTGCTC
GGAAGAGTATGAAGATGAACAAAGCCCTGAAAAGATTATCGAGCTGTATGCGGAGTGCATCAGGCTCTTTCACTCCATCGACATATCGGA
TTGTCCCTATACGAATAGCTTAGACAGCCGCTTAGCCGAATTGGATTACTTACTGAATAACGATCTGGCCGATGTGGATTGCGAAAACTG
GGAAGAAGACACTCCATTTAAAGATCCGCGCGAGCTGTATGATTTTTTAAAGACGGAAAAGCCCGAAGAGGAACTTGTCTTTTCCCACGG
CGACCTGGGAGACAGCAACATCTTTGTGAAAGATGGCAAAGTAAGTGGCTTTATTGATCTTGGGAGAAGCGGCAGGGCGGACAAGTGGTA
TGACATTGCCTTCTGCGTCCGGTCGATCAGGGAGGATATCGGGGAAGAACAGTATGTCGAGCTATTTTTTGACTTACTGGGGATCAAGCC
TGATTGGGAGAAAATAAAATATTATATTTTACTGGATGAATTGTTTTAGTACCTAGAATGCATGACCAAAATCCCTTAACGTGAGTTTTC
GTTCCACTGAGCGTCAGACCCCGTAGAAAAGATCAAAGGATCTTCTTGAGATCCTTTTTTTCTGCGCGTAATCTGCTGCTTGCAAACAAA
AAAACCACCGCTACCAGCGGTGGTTTGTTTGCCGGATCAAGAGCTACCAACTCTTTTTCCGAAGGTAACTGGCTTCAGCAGAGCGCAGAT
ACCAAATACTGTCCTTCTAGTGTAGCCGTAGTTAGGCCACCACTTCAAGAACTCTGTAGCACCGCCTACATACCTCGCTCTGCTAATCCT
GTTACCAGTGGCTGCTGCCAGTGGCGATAAGTCGTGTCTTACCGGGTTGGACTCAAGACGATAGTTACCGGATAAGGCGCAGCGGTCGGG
CTGAACGGGGGGTTCGTGCACACAGCCCAGCTTGGAGCGAACGACCTACACCGAACTGAGATACCTACAGCGTGAGCTATGAGAAAGCGC
CACGCTTCCCGAAGGGAGAAAGGCGGACAGGTATCCGGTAAGCGGCAGGGTCGGAACAGGAGAGCGCACGAGGGAGCTTCCAGGGGGAAA
CGCCTGGTATCTTTATAGTCCTGTCGGGTTTCGCCACCTCTGACTTGAGCGTCGATTTTTGTGATGCTCGTCAGGGGGGCGGAGCCTATG
GAAAAACGCCAGCAACGCGGCCTTTTTACGGTTCCTGGCCTTTTGCTGGCCTTTTGCTCACATGTTCTTTCCTGCGTTATCCCCTGATTC
TGTGGATAACCGTATTACCGCCTTTGAGTGAGCTGATACCGCTCGCCGCAGCCGAACGACCGAGCGCAGCGAGTCAGTGAGCGAGGAAGC
GGAAGAGCGCCTGATGCGGTATTTTCTCCTTACGCATCTGTGCGGTATTTCACACCGCATATGGTGCACTCTCAGTACAATCTGCTCTGA
TGCCGCATAGTTAAGCCAGTATACACTCCGCTATCGCTACGTGACTGGGTCATGGCTGCGCCCCGACACCCGCCAACACCCGCTGACGCG
CCCTGACGGGCTTGTCTGCTCCCGGCATCCGCTTACAGACAAGCTGTGACCGTCTCCGGGAGCTGCATGTGTCAGAGGTTTTCACCGTCA
TCACCGAAACGCGCGAGGCAGGGTGCCTTGATGTGGGCGCCGGCGGTCGAGTGGCGACGGCGCGGCTTGTCCGCGCCCTGGTAGATTGCC
TGGCCGTAGGCCAGCCATTTTTGAGCGGCCAGCGGCCGCGATAGGCCGACGCGAAGCGGCGGGGCGTAGGGAGCGCAGCGACCGAAGGGT
AGGCGCTTTTTGCAGCTCTTCGGCTGTGCGCTGGCCAGACAGTTATGCACAGGCCAGGCGGGTTTTAAGAGTTTTAATAAGTTTTAAAGA
GTTTTAGGCGGAAAAATCGCCTTTTTTCTCTTTTATATCAGTCACTTACATGTGTGACCGGTTCCCAATGTACGGCTTTGGGTTCCCAAT
GTACGGGTTCCGGTTCCCAATGTACGGCTTTGGGTTCCCAATGTACGTGCTATCCACAGGAAAGAGAACTTTTCGACCTTTTTCCCCTGC
TAGGGCAATTTGCCCTAGCATCTGCTCCGTACATTAGGAACCGGCGGATGCTTCGCCCTCGATCAGGTTGCGGTAGCGCATGACTAGGAT
CGGGCCAGCCTGCCCCGCCTCCTCCTTCAAATCGTACTCCGGCAGGTCATTTGACCCGATCAGCTTGCGCACGGTGAAACAGAACTTCTT
GAACTCTCCGGCGCTGCCACTGCGTTCGTAGATCGTCTTGAACAACCATCTGGCTTCTGCCTTGCCTGCGGCGCGGCGTGCCAGGCGGTA
GAGAAAACGGCCGATGCCGGGATCGATCAAAAAGTAATCGGGGTGAACCGTCAGCACGTCCGGGTTCTTGCCTTCTGTGATCTCGCGGTA
CATCCAATCAGCTAGCTCGATCTCGATGTACTCCGGCCGCCCGGTTTCGCTCTTTACGATCTTGTAGCGGCTAATCAAGGCTTCACCCTC
GGATACCGTCACCAGGCGGCCGTTCTTGGCCTTCTTCGTACGCTGCATGGCAACGTGCGTGGTGTTTAACCGAATGCAGGTTTCTACCAG
GTCGTCTTTCTGCTTTCCGCCATCGGCTCGCCGGCAGAACTTGAGTACGTCCGCAACGTGTGGACGGAACACGCGGCCGGGCTTGTCTCC
CTTCCCTTCCCGGTATCGGTTCATGGATTCGGTTAGATGGGAAACCGCCATCAGTACCAGGTCGTAATCCCACACACTGGCCATGCCGGC
CGGCCCTGCGGAAACCTCTACGTGCCCGTCTGGAAGCTCGTAGCGGATCACCTCGCCAGCTCGTCGGTCACGCTTCGACAGACGGAAAAC
GGCCACGTCCATGATGCTGCGACTATCGCGGGTGCCCACGTCATAGAGCATCGGAACGAAAAAATCTGGTTGCTCGTCGCCCTTGGGCGG
CTTCCTAATCGACGGCGCACCGGCTGCCGGCGGTTGCCGGGATTCTTTGCGGATTCGATCAGCGGCCGCTTGCCACGATTCACCGGGGCG
TGCTTCTGCCTCGATGCGTTGCCGCTGGGCGGCCTGCGCGGCCTTCAACTTCTCCACCAGGTCATCACCCAGCGCCGCGCCGATTTGTAC
CGGGCCGGATGGTTTGCGACCGTCACGCCGATTCCTCGGGCTTGGGGGTTCCAGTGCCATTGCAGGGCCGGCAGACAACCCAGCCGCTTA
CGCCTGGCCAACCGCCCGTTCCTCCACACATGGGGCATTCCACGGCGTCGGTGCCTGGTTGTTCTTGATTTTCCATGCCGCCTCCTTTAG
CCGCTAAAATTCATCTACTCATTTATTCATTTGCTCATTTACTCTGGTAGCTGCGCGATGTATTCAGATAGCAGCTCGGTAATGGTCTTG
CCTTGGCGTACCGCGTACATCTTCAGCTTGGTGTGATCCTCCGCCGGCAACTGAAAGTTGACCCGCTTCATGGCTGGCGTGTCTGCCAGG
CTGGCCAACGTTGCAGCCTTGCTGCTGCGTGCGCTCGGACGGCCGGCACTTAGCGTGTTTGTGCTTTTGCTCATTTTCTCTTTACCTCAT
TAACTCAAATGAGTTTTGATTTAATTTCAGCGGCCAGCGCCTGGACCTCGCGGGCAGCGTCGCCCTCGGGTTCTGATTCAAGAACGGTTG
TGCCGGCGGCGGCAGTGCCTGGGTAGCTCACGCGCTGCGTGATACGGGACTCAAGAATGGGCAGCTCGTACCCGGCCAGCGCCTCGGCAA
CCTCACCGCCGATGCGCGTGCCTTTGATCGCCCGCGACACGACAAAGGCCGCTTGTAGCCTTCCATCCGTGACCTCAATGCGCTGCTTAA
CCAGCTCCACCAGGTCGGCGGTGGCCCATATGTCGTAAGGGCTTGGCTGCACCGGAATCAGCACGAAGTCGGCTGCCTTGATCGCGGACA
CAGCCAAGTCCGCCGCCTGGGGCGCTCCGTCGATCACTACGAAGTCGCGCCGGCCGATGGCCTTCACGTCGCGGTCAATCGTCGGGCGGT
CGATGCCGACAACGGTTAGCGGTTGATCTTCCCGCACGGCCGCCCAATCGCGGGCACTGCCCTGGGGATCGGAATCGACTAACAGAACAT
CGGCCCCGGCGAGTTGCAGGGCGCGGGCTAGATGGGTTGCGATGGTCGTCTTGCCTGACCCGCCTTTCTGGTTAAGTACAGCGATAACCT
TCATGCGTTCCCCTTGCGTATTTGTTTATTTACTCATCGCATCATATACGCAGCGACCGCATGACGCAAGCTGTTTTACTCAAATACACA
TCACCTTTTTAGACGGCGGCGCTCGGTTTCTTCAGCGGCCAAGCTGGCCGGCCAGGCCGCCAGCTTGGCATCAGACAAACCGGCCAGGAT
TTCATGCAGCCGCACGGTTGAGACGTGCGCGGGCGGCTCGAACACGTACCCGGCCGCGATCATCTCCGCCTCGATCTCTTCGGTAATGAA
AAACGGTTCGTCCTGGCCGTCCTGGTGCGGTTTCATGCTTGTTCCTCTTGGCGTTCATTCTCGGCGGCCGCCAGGGCGTCGGCCTCGGTC
AATGCGTCCTCACGGAAGGCACCGCGCCGCCTGGCCTCGGTGGGCGTCACTTCCTCGCTGCGCTCAAGTGCGCGGTACAGGGTCGAGCGA
TGCACGCCAAGCAGTGCAGCCGCCTCTTTCACGGTGCGGCCTTCCTGGTCGATCAGCTCGCGGGCGTGCGCGATCTGTGCCGGGGTGAGG
GTAGGGCGGGGGCCAAACTTCACGCCTCGGGCCTTGGCGGCCTCGCGCCCGCTCCGGGTGCGGTCGATGATTAGGGAACGCTCGAACTCG

GCAATGCCGGCGAACACGGTCAACACCATGCGGCCGGCCGGCGTGGTGGTGTCGGCCCACGGCTCTGCCAGGCTACGCAGGCCCGCGCCG
GCCTCCTGGATGCGCTCGGCAATGTCCAGTAGGTCGCGGGTGCTGCGGGCCAGGCGGTCTAGCCTGGTCACTGTCACAACGTCGCCAGGG
CGTAGGTGGTCAAGCATCCTGGCCAGCTCCGGGCGGTCGCGCCTGGTGCCGGTGATCTTCTCGGAAAACAGCTTGGTGCAGCCGGCCGCG
TGCAGTTCGGCCCGTTGGTTGGTCAAGTCCTGGTCGTCGGTGCTGACGCGGGCATAGCCCAGCAGGCCAGCGGCGGCGCTCTTGTTCATG
GCGTAATGTCTCCGGTTCTAGTCGCAAGTATTCTACTTTATGCGACTAAAACACGCGACAAGAAAACGCCAGGAAAAGGGCAGGGCGGCA
GCCTGTCGCGTAACTTAGGACTTGTGCGACATGTCGTTTTCAGAAGACGGCTGCACTGAACGTCAGAAGCCGACTGCACTATAGCAGCGG
AGGGGTTGGATCAAAGTACTTTGATCCCGAGGGGAACCCTGTGGTTGGCATGCACATACAAATGGACGAACGGATAAACCTTTTCACGCC
CTTTTAAATATCCGTTATTCTAATAAACGCTCTTTTCTCTTAGGTTTACCCGCCAATATATCCTGTCAAACACTGATAGTTTAAACTGAA
GGCGGGAAACGACAATCTGATCCAAGCTCAAGCTGCTCTAGCATTCGCCATTCAGGCTGCGCAACTGTTGGGAAGGGCGATCGGTGCGGG
CCTCTTCGCTATTACGCCAGCTGGCGAAAGGGGGATGTGCTGCAAGGCGATTAAGTTGGGTAACGCCAGGGTTTTCCCAGTCACGACGTT
GTAAAACGACGGCCAGTGCCAAGCTTGGCGTGCCTGCAGGTCAACATGGTGGAGCACGACACACTTGTCTACTCCAAAAATATCAAAGAT
ACAGTCTCAGAAGACCAAAGGGCAATTGAGACTTTTCAACAAAGGGTAATATCCGGAAACCTCCTCGGATTCCATTGCCCAGCTATCTGT
CACTTTATTGTGAAGATAGTGGAAAAGGAAGGTGGCTCCTACAAATGCCATCATTGCGATAAAGGAAAGGCCATCGTTGAAGATGCCTCT
GCCGACAGTGGTCCCAAAGATGGACCCCCACCCACGAGGAGCATCGTGGAAAAAGAAGACGTTCCAACCACGTCTTCAAAGCAAGTGGAT
TGATGTGATAACATGGTGGAGCACGACACACTTGTCTACTCCAAAAATATCAAAGATACAGTCTCAGAAGACCAAAGGGCAATTGAGACT
TTTCAACAAAGGGTAATATCCGGAAACCTCCTCGGATTCCATTGCCCAGCTATCTGTCACTTTATTGTGAAGATAGTGGAAAAGGAAGGT
GGCTCCTACAAATGCCATCATTGCGATAAAGGAAAGGCCATCGTTGAAGATGCCTCTGCCGACAGTGGTCCCAAAGATGGACCCCCACCC
ACGAGGAGCATCGTGGAAAAAGAAGACGTTCCAACCACGTCTTCAAAGCAAGTGGATTGATGTGATATCTCCACTGACGTAAGGGATGAC
GCACAATCCCACTATCCTTCGCAAGACCCTTCCTCTATATAAGGAAGTTCATTTCATTTGGAGAGGACCTCGACTCTAGAGGATCCCCGG
GTACCGGGCCCCCCCTCGAGGCGCGCCAAGCTATCAAACAAGTTTGTACAAAAAAGCAGGCTCCGCGGCCGCCCCCTTCACCTGTA**GTGA**
TTTTTCTCTACAAGCGAATAGACCATTTAAGAGACCATTAGGCACCCCAGGCTTTACACTTTATGCTTCCGGCTCGTATAATGTGTGGAT
TTTGAGTTAGGAGCCGTCGAGATTTTCAGGAGCTAAGGAAGCTAAAATGGAGAAAAAAATCACTGGATATACCACCGTTGATATATCCCA
ATGGCATCGTAAAGAACATTTTGAGGCATTTCAGTCAGTTGCTCAATGTACCTATAACCAGACCGTTCAGCTGGATATTACGGCCTTTTT
AAAGACCGTAAAGAAAAATAAGCACAAGTTTTATCCGGCCTTTATTCACATTCTTGCCCGCCTGATGAATGCTCATCCGGAGTTCCGTAT
GGCAATGAAAGACGGTGAGCTGGTGATATGGGATAGTGTTCACCCTTGTTACACCGTTTTCCATGAGCAAACTGAAACGTTTTCATCGCT
CTGGAGTGAATACCACGACGATTTCCGGCAGTTTCTACACATATATTCGCAAGATGTGGCGTGTTACGGTGAAAACCTGGCCTATTTCCC
TAAAGGGTTTATTGAGAATATGTTTTTCGTCTCAGCCAATCCCTGGGTGAGTTTCACCAGTTTTGATTTAAACGTGGCCAATATGGACAA
CTTCTTCGCCCCCGTTTTCACCATGGGCAAATATTATACGCAAGGCGACAAGGTGCTGATGCCGCTGGCGATTCAGGTTCATCATGCCGT
TTGTGATGGCTTCCATGTCGGCAGAATGCTTAATGAATTACAACAGTACTGCGATGAGTGGCAGGGCGGGGCGTAAACGCGTGGAGCCGG
CTTACTAAAAGCCAGATAACAGTATGCGTATTTGCGCGCTGATTTTTGCGGTATAAGAATATATACTGATATGTATACCCGAAGTATGTC
AAAAAGAGGTATGCTATGAAGCAGCGTATTACAGTGACAGTTGACAGCGACAGCTATCAGTTGCTCAAGGCATATATGATGTCAATATCT
CCGGTCTGGTAAGCACAACCATGCAGAATGAAGCCCGTCGTCTGCGTGCCGAACGCTGGAAAGCGGAAAATCAGGAAGGGATGGCTGAGG
TCGCCCGGTTTATTGAAATGAACGGCTCTTTTGCTGACGAGAACAGGGGCTGGTGAAATGCAGTTTAAGGTTTACACCTATAAAAGAGAG
AGCCGTTATCGTCTGTTTGTGGATGTACAGAGTGATATTATTGACACGCCCGGCCGACGGATGGTGATCCCCCTGGCCAGTGCACGTCTG
CTGTCAGATAAAGTCTCCCGTGAACTTTACCCGGTGGTGCATATCGGGGATGAAAGCTGGCGCATGATGACCACCGATATGGCCAGTGTG
CCGGTTTCCGTTATCGGGGAAGAAGTGGCTGATCTCAGCCACCGCGAAAATGACATCAAAAACGCCATTAACCTGATGTTCTGGGGAATA
TAAATGTCAGGCTCCCTTATACACAGCCAGTCTGCACCTCGACGGTCTCACATTAAGGGTGGGCGCGCCGACCCAGCTTTCTTGTACAAA
GTGGTTCGATAATTCCTTAATTAACTAGTTCTAGAGCGGCCGCCCACCGCGGTGGAGCTCGAATTTCCCCGATCGTTCAAACATTTGGCA
ATAAAGTTTCTTAAGATTGAATCCTGTTGCCGGTCTTGCGATGATTATCATATAATTTCTGTTGAATTACGTTAAGCATGTAATAATTAA
CATGTAATGCATGACGTTATTTATGAGATGGGTTTTTATGATTAGAGTCCCGCAATTATACATTTAATACGCGATAGAAAACAAAATATA
GCGCGCAAACTAGGATAAATTATCGCGCGCGGTGTCATCTATGTTACTGAATTCGTAATCATGGTCATAGCTGTTTCCTGTGTGAAATTG
TTATCCGCTCACAATTCCACACAACATACGAGCCGGAAGCATAAAGTGTAAAGCCTGGGGTGCCTAATGAGTGAGCTAACTCACATTAAT
TGCGTTGCGCTCACTGCCCGCTTTCCAGTCGGGAAACCTGTCGTGCCAGCTGCATTAATGAATCGGCCAACGCGCGGGGAGAGGCGGTTT
GCGTATTGGCTAGAGCAGCTTGCCAACATGGTGGAGCACGACACTCTCGTCTACTCCAAGAATATCAAAGATACAGTCTCAGAAGACCAA
AGGGCTATTGAGACTTTTCAACAAAGGGTAATATCGGGAAACCTCCTCGGATTCCATTGCCCAGCTATCTGTCACTTCATCAAAAGGACA
GTAGAAAAGGAAGGTGGCACCTACAAATGCCATCATTGCGATAAAGGAAAGGCTATCGTTCAAGATGCCTCTGCCGACAGTGGTCCCAAA
GATGGACCCCCACCCACGAGGAGCATCGTGGAAAAAGAAGACGTTCCAACCACGTCTTCAAAGCAAGTGGATTGATGTGATAACATGGTG
GAGCACGACACTCTCGTCTACTCCAAGAATATCAAAGATACAGTCTCAGAAGACCAAAGGGCTATTGAGACTTTTCAACAAAGGGTAATA
TCGGGAAACCTCCTCGGATTCCATTGCCCAGCTATCTGTCACTTCATCAAAAGGACAGTAGAAAAGGAAGGTGGCACCTACAAATGCCAT
CATTGCGATAAAGGAAAGGCTATCGTTCAAGATGCCTCTGCCGACAGTGGTCCCAAAGATGGACCCCCACCCACGAGGAGCATCGTGGAA
AAAGAAGACGTTCCAACCACGTCTTCAAAGCAAGTGGATTGATGTGATATCTCCACTGACGTAAGGGATGACGCACAATCCCACTATCCT
TCGCAAGACCTTCCTCTATATAAGGAAGTTCATTTCATTTGGAGAGGACACGCTGAAATCACCAGTCTCTCTCTACAAATCTATCTCTCT
CGAGCTTTCGCAGATCCCGGGGGGCAATGAGATATGAAAAAGCCTGAACTCACCGCGACGTCTGTCGAGAAGTTTCTGATCGAAAAGTTC
GACAGCGTCTCCGACCTGATGCAGCTCTCGGAGGGCGAAGAATCTCGTGCTTTCAGCTTCGATGTAGGAGGGCGTGGATATGTCCTGCGG
GTAAATAGCTGCGCCGATGGTTTCTACAAAGATCGTTATGTTTATCGGCACTTTGCATCGGCCGCGCTCCCGATTCCGGAAGTGCTTGAC
ATTGGGGAGTTTAGCGAGAGCCTGACCTATTGCATCTCCCGCCGTGCACAGGGTGTCACGTTGCAAGACCTGCCTGAAACCGAACTGCCC
GCTGTTCTACAACCGGTCGCGGAGGCTATGGATGCGATCGCTGCGGCCGATCTTAGCCAGACGAGCGGGTTCGGCCCATTCGGACCGCAA
GGAATCGGTCAATACACTACATGGCGTGATTTCATATGCGCGATTGCTGATCCCCATGTGTATCACTGGCAAACTGTGATGGACGACACC
GTCAGTGCGTCCGTCGCGCAGGCTCTCGATGAGCTGATGCTTTGGGCCGAGGACTGCCCCGAAGTCCGGCACCTCGTGCACGCGGATTTC
GGCTCCAACAATGTCCTGACGGACAATGGCCGCATAACAGCGGTCATTGACTGGAGCGAGGCGATGTTCGGGGATTCCCAATACGAGGTC
GCCAACATCTTCTTCTGGAGGCCGTGGTTGGCTTGTATGGAGCAGCAGACGCGCTACTTCGAGCGGAGGCATCCGGAGCTTGCAGGATCG
CCACGACTCCGGGCGTATATGCTCCGCATTGGTCTTGACCAACTCTATCAGAGCTTGGTTGACGGCAATTTCGATGATGCAGCTTGGGCG
CAGGGTCGATGCGACGCAATCGTCCGATCCGGAGCCGGGACTGTCGGGCGTACACAAATCGCCCGCAGAAGCGCGGCCGTCTGGACCGAT

GGCTGTGTAGAAGTACTCGCCGATAGTGGAAACCGACGCCCCAGCACTCGTCCGAGGGCAAAGAAATAGAGTAGATGCCGACCGGATCTG
TCGATCGACAAGCTCGAGTTTCTCCATAATAATGTGTGAGTAGTTCCCAGATAAGGGAATTAGGGTTCCTATAGGGTTTCGCTCATGTGT
TGAGCATATAAGAAACCCTTAGTATGTATTTGTATTTGTAAAATACTTCTATCAATAAAATTTCTAATTCCTAAAACCAAAATCCAGTAC
TAAAATCCAGATCCCCCGAATTAATTCGGCGTTAATTCAGTACATTAAAAACGTCCGCAATGTGTTATTAAGTTGTCTAAGCGTCAATTT
GTTTACACCACAATATATCCTGCCA

AtmiR173a target site

AtTAS1c-derived spacer

T-DNA right border

T-DNA left border

*ccd*B gene

*Bsa*I site

Inverted *Bsa*I site

Chloramphenicol resistance gene

attB1

attB2

Nos terminator

CaMV promoter

kanamycin resistance gene

Hygromycin resistance gene

2x35S CaMV promoter

CaMV terminator

>pMDC32B-NbmiR482aTS-B/c (11635 bp)

CCAGCCAGCCAACAGCTCCCCGACCGGCAGCTCGGCACAAAATCACCACTCGATACAGGCAGCCCATCAGTCCGGGACGGCGTCAGCGGG
AGAGCCGTTGTAAGGCGGCAGACTTTGCTCATGTTACCGATGCTATTCGGAAGAACGGCAACTAAGCTGCCGGGTTTGAAACACGGATGA
TCTCGCGGAGGGTAGCATGTTGATTGTAACGATGACAGAGCGTTGCTGCCTGTGATCACCGCGGTTTCAAAATCGGCTCCGTCGATACTA
TGTTATACGCCAACTTTGAAAACAACTTTGAAAAAGCTGTTTTCTGGTATTTAAGGTTTTAGAATGCAAGGAACAGTGAATTGGAGTTCG
TCTTGTTATAATTAGCTTCTTGGGGTATCTTTAAATACTGTAGAAAAGAGGAAGGAAATAATAAATGGCTAAAATGAGAATATCACCGGA
ATTGAAAAAACTGATCGAAAAATACCGCTGCGTAAAAGATACGGAAGGAATGTCTCCTGCTAAGGTATATAAGCTGGTGGGAGAAAATGA
AAACCTATATTTAAAAATGACGGACAGCCGGTATAAAGGGACCACCTATGATGTGGAACGGGAAAAGGACATGATGCTATGGCTGGAAGG
AAAGCTGCCTGTTCCAAAGGTCCTGCACTTTGAACGGCATGATGGCTGGAGCAATCTGCTCATGAGTGAGGCCGATGGCGTCCTTTGCTC
GGAAGAGTATGAAGATGAACAAAGCCCTGAAAAGATTATCGAGCTGTATGCGGAGTGCATCAGGCTCTTTCACTCCATCGACATATCGGA
TTGTCCCTATACGAATAGCTTAGACAGCCGCTTAGCCGAATTGGATTACTTACTGAATAACGATCTGGCCGATGTGGATTGCGAAAACTG
GGAAGAAGACACTCCATTTAAAGATCCGCGCGAGCTGTATGATTTTTTAAAGACGGAAAAGCCCGAAGAGGAACTTGTCTTTTCCCACGG
CGACCTGGGAGACAGCAACATCTTTGTGAAAGATGGCAAAGTAAGTGGCTTTATTGATCTTGGGAGAAGCGGCAGGGCGGACAAGTGGTA
TGACATTGCCTTCTGCGTCCGGTCGATCAGGGAGGATATCGGGGAAGAACAGTATGTCGAGCTATTTTTTGACTTACTGGGGATCAAGCC
TGATTGGGAGAAAATAAAATATTATATTTTACTGGATGAATTGTTTTAGTACCTAGAATGCATGACCAAAATCCCTTAACGTGAGTTTTC
GTTCCACTGAGCGTCAGACCCCGTAGAAAAGATCAAAGGATCTTCTTGAGATCCTTTTTTTCTGCGCGTAATCTGCTGCTTGCAAACAAA
AAAACCACCGCTACCAGCGGTGGTTTGTTTGCCGGATCAAGAGCTACCAACTCTTTTTCCGAAGGTAACTGGCTTCAGCAGAGCGCAGAT
ACCAAATACTGTCCTTCTAGTGTAGCCGTAGTTAGGCCACCACTTCAAGAACTCTGTAGCACCGCCTACATACCTCGCTCTGCTAATCCT
GTTACCAGTGGCTGCTGCCAGTGGCGATAAGTCGTGTCTTACCGGGTTGGACTCAAGACGATAGTTACCGGATAAGGCGCAGCGGTCGGG
CTGAACGGGGGGTTCGTGCACACAGCCCAGCTTGGAGCGAACGACCTACACCGAACTGAGATACCTACAGCGTGAGCTATGAGAAAGCGC
CACGCTTCCCGAAGGGAGAAAGGCGGACAGGTATCCGGTAAGCGGCAGGGTCGGAACAGGAGAGCGCACGAGGGAGCTTCCA GGGGGAAA
CGCCTGGTATCTTTATAGTCCTGTCGGGTTTCGCCACCTCTGACTTGAGCGTCGATTTTTGTGATGCTCGTCAGGGGGGCGGAGCCTATG
GAAAAACGCCAGCAACGCGGCCTTTTTACGGTTCCTGGCCTTTTGCTGGCCTTTTGCTCACATGTTCTTTCCTGCGTTATCCCCTGATTC
TGTGGATAACCGTATTACCGCCTTTGAGTGAGCTGATACCGCTCGCCGCAGCCGAACGACCGAGCGCAGCGAGTCAGTGAGCGAGGAAGC
GGAAGAGCGCCTGATGCGGTATTTTCTCCTTACGCATCTGTGCGGTATTTCACACCGCATATGGTGCACTCTCAGTACAATCTGCTCTGA
TGCCGCATAGTTAAGCCAGTATACACTCCGCTATCGCTACGTGACTGGGTCATGGCTGCGCCCCGACACCCGCCAACACCCGCTGACGCG
CCCTGACGGGCTTGTCTGCTCCCGGCATCCGCTTACAGACAAGCTGTGACCGTCTCCGGGAGCTGCATGTGTCAGAGGTTTTCACCGTCA
TCACCGAAACGCGCGAGGCAGGGTGCCTTGATGTGGGCGCCGGCGGTCGAGTGGCGACGGCGCGGCTTGTCCGCGCCCTGGTAGATTGCC
TGGCCGTAGGCCAGCCATTTTTGAGCGGCCAGCGGCCGCGATAGGCCGACGCGAAGCGGCGGGGCGTAGGGAGCGCAGCGACCGAAGGGT
AGGCGCTTTTTGCAGCTCTTCGGCTGTGCGCTGGCCAGACAGTTATGCACAGGCCAGGCGGGTTTTAAGAGTTTTAATAAGTTTTAAAGA
GTTTTAGGCGGAAAAATCGCCTTTTTTCTCTTTTATATCAGTCACTTACATGTGTGACCGGTTCCCAATGTACGGCTTTGGGTTCCCAAT
GTACGGGTTCCGGTTCCCAATGTACGGCTTTGGGTTCCCAATGTACGTGCTATCCACAGGAAAGAGAACTTTTCGACCTTTTTCCCCTGC
TAGGGCAATTTGCCCTAGCATCTGCTCCGTACATTAGGAACCGGCGGATGCTTCGCCCTCGATCAGGTTGCGGTAGCGCATGACTAGGAT
CGGGCCAGCCTGCCCCGCCTCCTCCTTCAAATCGTACTCCGGCAGGTCATTTGACCCGATCAGCTTGCGCACGGTGAAACAGAACTTCTT
GAACTCTCCGGCGCTGCCACTGCGTTCGTAGATCGTCTTGAACAACCATCTGGCTTCTGCCTTGCCTGCGGCGCGGCGTGCCAGGCGGTA
GAGAAAACGGCCGATGCCGGGATCGATCAAAAAGTAATCGGGGTGAACCGTCAGCACGTCCGGGTTCTTGCCTTCTGTGATCTCGCGGTA
CATCCAATCAGCTAGCTCGATCTCGATGTACTCCGGCCGCCCGGTTTCGCTCTTTACGATCTTGTAGCGGCTAATCAAGGCTTCACCCTC
GGATACCGTCACCAGGCGGCCGTTCTTGGCCTTCTTCGTACGCTGCATGGCAACGTGCGTGGTGTTTAACCGAATGCAGGTTTCTACCAG
GTCGTCTTTCTGCTTTCCGCCATCGGCTCGCCGGCAGAACTTGAGTACGTCCGCAACGTGTGGACGGAACACGCGGCCGGGCTTGTCTCC
CTTCCCTTCCCGGTATCGGTTCATGGATTCGGTTAGATGGGAAACCGCCATCAGTACCAGGTCGTAATCCCACACACTGGCCATGCCGGC
CGGCCCTGCGGAAACCTCTACGTGCCCGTCTGGAAGCTCGTAGCGGATCACCTCGCCAGCTCGTCGGTCACGCTTCGACAGACGGAAAAC
GGCCACGTCCATGATGCTGCGACTATCGCGGGTGCCCACGTCATAGAGCATCGGAACGAAAAAATCTGGTTGCTCGTCGCCCTTGGGCGG
CTTCCTAATCGACGGCGCACCGGCTGCCGGCGGTTGCCGGGATTCTTTGCGGATTCGATCAGCGGCCGCTTGCCACGATTCACCGGGGCG
TGCTTCTGCCTCGATGCGTTGCCGCTGGGCGGCCTGCGCGGCCTTCAACTTCTCCACCAGGTCATCACCCAGCGCCGCGCCGATTTGTAC
CGGGCCGGATGGTTTGCGACCGTCACGCCGATTCCTCGGGCTTGGGGGTTCCAGTGCCATTGCAGGGCCGGCAGACAACCCAGCCGCTTA
CGCCTGGCCAACCGCCCGTTCCTCCACACATGGGGCATTCCACGGCGTCGGTGCCTGGTTGTTCTTGATTTTCCATGCCGCCTCCTTTAG
CCGCTAAAATTCATCTACTCATTTATTCATTTGCTCATTTACTCTGGTAGCTGCGCGATGTATTCAGATAGCAGCTCGGTAATGGTCTTG
CCTTGGCGTACCGCGTACATCTTCAGCTTGGTGTGATCCTCCGCCGGCAACTGAAAGTTGACCCGCTTCATGGCTGGCGTGTCTGCCAGG
CTGGCCAACGTTGCAGCCTTGCTGCTGCGTGCGCTCGGACGGCCGGCACTTAGCGTGTTTGTGCTTTTGCTCATTTTCTCTTTACCTCAT
TAACTCAAATGAGTTTTGATTTAATTTCAGCGGCCAGCGCCTGGACCTCGCGGGCAGCGTCGCCCTCGGGTTCTGATTCAAGAACGGTTG
TGCCGGCGGCGGCAGTGCCTGGGTAGCTCACGCGCTGCGTGATACGGGACTCAAGAATGGGCAGCTCGTACCCGGCCAGCGCCTCGGCAA
CCTCACCGCCGATGCGCGTGCCTTTGATCGCCCGCGACACGACAAAGGCCGCTTGTAGCCTTCCATCCGTGACCTCAATGCGCTGCTTAA
CCAGCTCCACCAGGTCGGCGGTGGCCCATATGTCGTAAGGGCTTGGCTGCACCGGAATCAGCACGAAGTCGGCTGCCTTGATCGCGGACA
CAGCCAAGTCCGCCGCCTGGGGCGCTCCGTCGATCACTACGAAGTCGCGCCGGCCGATGGCCTTCACGTCGCGGTCAATCGTCGGGCGGT
CGATGCCGACAACGGTTAGCGGTTGATCTTCCCGCACGGCCGCCCAATCGCGGGCACTGCCCTGGGGATCGGAATCGACTAACAGAACAT
CGGCCCCGGCGAGTTGCAGGGCGCGGGCTAGATGGGTTGCGATGGTCGTCTTGCCTGACCCGCCTTTCTGGTTAAGTACAGCGATAACCT
TCATGCGTTCCCCTTGCGTATTTGTTTATTTACTCATCGCATCATATACGCAGCGACCGCATGACGCAAGCTGTTTTACTCAAATACACA
TCACCTTTTTAGACGGCGGCGCTCGGTTTCTTCAGCGGCCAAGCTGGCCGGCCAGGCCGCCAGCTTGGCATCAGACAAAC CGGCCAGGAT
TTCATGCAGCCGCACGGTTGAGACGTGCGCGGGCGGCTCGAACACGTACCCGGCCGCGATCATCTCCGCCTCGATCTCTTCGGTAATGAA
AAACGGTTCGTCCTGGCCGTCCTGGTGCGGTTTCATGCTTGTTCCTCTTGGCGTTCATTCTCGGCGGCCGCCAGGGCGTCGGCCTCGGTC
AATGCGTCCTCACGGAAGGCACCGCGCCGCCTGGCCTCGGTGGGCGTCACTTCCTCGCTGCGCTCAAGTGCGCGGTACAGGGTCGAGCGA
TGCACGCCAAGCAGTGCAGCCGCCTCTTTCACGGTGCGGCCTTCCTGGTCGATCAGCTCGCGGGCGTGCGCGATCTGTGCCGGGGTGAGG
GTAGGGCGGGGGCCAAACTTCACGCCTCGGGCCTTGGCGGCCTCGCGCCCGCTCCGGGTGCGGTCGATGATTAGGGAACGCTCGAACTCG

GCAATGCCGGCGAACACGGTCAACACCATGCGGCCGGCCGGCGTGGTGGTGTCGGCCCACGGCTCTGCCAGGCTACGCAGGCCCGCGCCG
GCCTCCTGGATGCGCTCGGCAATGTCCAGTAGGTCGCGGGTGCTGCGGGCCAGGCGGTCTAGCCTGGTCACTGTCACAACGTCGCCAGGG
CGTAGGTGGTCAAGCATCCTGGCCAGCTCCGGGCGGTCGCGCCTGGTGCCGGTGATCTTCTCGGAAAACAGCTTGGTGCAGCCGGCCGCG
TGCAGTTCGGCCCGTTGGTTGGTCAAGTCCTGGTCGTCGGTGCTGACGCGGGCATAGCCCAGCAGGCCAGCGGCGGCGCTCTTGTTCATG
GCGTAATGTCTCCGGTTCTAGTCGCAAGTATTCTACTTTATGCGACTAAAACACGCGACAAGAAAACGCCAGGAAAAGGGCAGGGCGGCA
GCCTGTCGCGTAACTTAGGACTTGTGCGACATGTCGTTTTCAGAAGACGGCTGCACTGAACGTCAGAAGCCGACTGCACTATAGCAGCGG
AGGGGTTGGATCAAAGTACTTTGATCCCGAGGGGAACCCTGTGGTTGGCATGCACATACAAATGGACGAACGGATAAACCTTTTCACGCC
CTTTTAAATATCCGTTATTCTAATAAACGCTCTTTTCTCTTAGGTTTACCCGCCAATATATCCTGTCAAACACTGATAGTTTAAACTGAA
GGCGGGAAACGACAATCTGATCCAAGCTCAAGCTGCTCTAGCATTCGCCATTCAGGCTGCGCAACTGTTGGGAAGGGCGATCGGTGCGGG
CCTCTTCGCTATTACGCCAGCTGGCGAAAGGGGGATGTGCTGCAAGGCGATTAAGTTGGGTAACGCCAGGGTTTTCCCAGTCACGACGTT
GTAAAACGACGGCCAGTGCCAAGCTTGGCGTGCCTGCAGGTCAACATGGTGGAGCACGACACACTTGTCTACTCCAAAAATATCAAAGAT
ACAGTCTCAGAAGACCAAAGGGCAATTGAGACTTTTCAACAAAGGGTAATATCCGGAAACCTCCTCGGATTCCATTGCCCAGCTATCTGT
CACTTTATTGTGAAGATAGTGGAAAAGGAAGGTGGCTCCTACAAATGCCATCATTGCGATAAAGGAAAGGCCATCGTTGAAGATGCCTCT
GCCGACAGTGGTCCCAAAGATGGACCCCCACCCACGAGGAGCATCGTGGAAAAAGAAGACGTTCCAACCACGTCTTCAAAGCAAGTGGAT
TGATGTGATAACATGGTGGAGCACGACACACTTGTCTACTCCAAAAATATCAAAGATACAGTCTCAGAAGACCAAAGGGCAATTGAGACT
TTTCAACAAAGGGTAATATCCGGAAACCTCCTCGGATTCCATTGCCCAGCTATCTGTCACTTTATTGTGAAGATAGTGGAAAAGGAAGGT
GGCTCCTACAAATGCCATCATTGCGATAAAGGAAAGGCCATCGTTGAAGATGCCTCTGCCGACAGTGGTCCCAAAGATGGACCCCCACCC
ACGAGGAGCATCGTGGAAAAAGAAGACGTTCCAACCACGTCTTCAAAGCAAGTGGATTGATGTGATATCTCCACTGACGTAAGGGATGAC
GCACAATCCCACTATCCTTCGCAAGACCCTTCCTCTATATAAGGAAGTTCATTTCATTTGGAGAGGACCTCGACTCTAGAGGATCCCCGG
GTACCGGGCCCCCCCTCGAGGCGCGCCAAGCTATCAAACAAGTTTGTACAAAAAAGCAGGCTCCGCGGCCGCCCCCTTCACCTGTA**GTGG**
TATGGGGGGAGTCGGGAATAGACCATTTAAGAGACCATTAGGCACCCCAGGCTTTACACTTTATGCTTCCGGCTCGTATAATGTGTGGAT
TTTGAGTTAGGAGCCGTCGAGATTTTCAGGAGCTAAGGAAGCTAAAATGGAGAAAAAAATCACTGGATATACCACCGTTGATATATCCCA
ATGGCATCGTAAAGAACATTTTGAGGCATTTCAGTCAGTTGCTCAATGTACCTATAACCAGACCGTTCAGCTGGATATTACGGCCTTTTT
AAAGACCGTAAAGAAAAATAAGCACAAGTTTTATCCGGCCTTTATTCACATTCTTGCCCGCCTGATGAATGCTCATCCGGAGTTCCGTAT
GGCAATGAAAGACGGTGAGCTGGTGATATGGGATAGTGTTCACCCTTGTTACACCGTTTTCCATGAGCAAACTGAAACGTTTTCATCGCT
CTGGAGTGAATACCACGACGATTTCCGGCAGTTTCTACACATATATTCGCAAGATGTGGCGTGTTACGGTGAAAACCTGGCCTATTTCCC
TAAAGGGTTTATTGAGAATATGTTTTTCGTCTCAGCCAATCCCTGGGTGAGTTTCACCAGTTTTGATTTAAACGTGGCCAATATGGACAA
CTTCTTCGCCCCCGTTTTCACCATGGGCAAATATTATACGCAAGGCGACAAGGTGCTGATGCCGCTGGCGATTCAGGTTCATCATGCCGT
TTGTGATGGCTTCCATGTCGGCAGAATGCTTAATGAATTACAACAGTACTGCGATGAGTGGCAGGGCGGGGCGTAAACGCGTGGAGCCGG
CTTACTAAAAGCCAGATAACAGTATGCGTATTTGCGCGCTGATTTTTGCGGTATAAGAATATATACTGATATGTATACCCGAAGTATGTC
AAAAAGAGGTATGCTATGAAGCAGCGTATTACAGTGACAGTTGACAGCGACAGCTATCAGTTGCTCAAGGCATATATGATGTCAATATCT
CCGGTCTGGTAAGCACAACCATGCAGAATGAAGCCCGTCGTCTGCGTGCCGAACGCTGGAAAGCGGAAAATCAGGAAGGGATGGCTGAGG
TCGCCCGGTTTATTGAAATGAACGGCTCTTTTGCTGACGAGAACAGGGGCTGGTGAAATGCAGTTTAAGGTTTACACCTATAAAAGAGAG
AGCCGTTATCGTCTGTTTGTGGATGTACAGAGTGATATTATTGACACGCCCGGCCGACGGATGGTGATCCCCCTGGCCAGTGCACGTCTG
CTGTCAGATAAAGTCTCCCGTGAACTTTACCCGGTGGTGCATATCGGGGATGAAAGCTGGCGCATGATGACCACCGATATGGCCAGTGTG
CCGGTTTCCGTTATCGGGGAAGAAGTGGCTGATCTCAGCCACCGCGAAAATGACATCAAAAACGCCATTAACCTGATGTTCTGGGGAATA
TAAATGTCAGGCTCCCTTATACACAGCCAGTCTGCACCTCGACGGTCTCACATTAAGGGTGGGCGCGCCGACCCAGCTTTCTTGTACAAA
GTGGTTCGATAATTCCTTAATTAACTAGTTCTAGAGCGGCCGCCCACCGCGGTGGAGCTCGAATTTCCCCGATCGTTCAAACATTTGGCA
ATAAAGTTTCTTAAGATTGAATCCTGTTGCCGGTCTTGCGATGATTATCATATAATTTCTGTTGAATTACGTTAAGCATGTAATAATTAA
CATGTAATGCATGACGTTATTTATGAGATGGGTTTTTATGATTAGAGTCCCGCAATTATACATTTAATACGCGATAGAAAACAAAATATA
GCGCGCAAACTAGGATAAATTATCGCGCGCGGTGTCATCTATGTTACTGAATTCGTAATCATGGTCATAGCTGTTTCCTGTGTGAAATTG
TTATCCGCTCACAATTCCACACAACATACGAGCCGGAAGCATAAAGTGTAAAGCCTGGGGTGCCTAATGAGTGAGCTAACTCACATTAAT
TGCGTTGCGCTCACTGCCCGCTTTCCAGTCGGGAAACCTGTCGTGCCAGCTGCATTAATGAATCGGCCAACGCGCGGGGAGAGGCGGTTT
GCGTATTGGCTAGAGCAGCTTGCCAACATGGTGGAGCACGACACTCTCGTCTACTCCAAGAATATCAAAGATACAGTCTCAGAAGACCAA
AGGGCTATTGAGACTTTTCAACAAAGGGTAATATCGGGAAACCTCCTCGGATTCCATTGCCCAGCTATCTGTCACTTCATCAAAAGGACA
GTAGAAAAGGAAGGTGGCACCTACAAATGCCATCATTGCGATAAAGGAAAGGCTATCGTTCAAGATGCCTCTGCCGACAGTGGTCCCAAA
GATGGACCCCCACCCACGAGGAGCATCGTGGAAAAAGAAGACGTTCCAACCACGTCTTCAAAGCAAGTGGATTGATGTGATAACATGGTG
GAGCACGACACTCTCGTCTACTCCAAGAATATCAAAGATACAGTCTCAGAAGACCAAAGGGCTATTGAGACTTTTCAACAAAGGGTAATA
TCGGGAAACCTCCTCGGATTCCATTGCCCAGCTATCTGTCACTTCATCAAAAGGACAGTAGAAAAGGAAGGTGGCACCTACAAATGCCAT
CATTGCGATAAAGGAAAGGCTATCGTTCAAGATGCCTCTGCCGACAGTGGTCCCAAAGATGGACCCCCACCCACGAGGAGCATCGTGGAA
AAAGAAGACGTTCCAACCACGTCTTCAAAGCAAGTGGATTGATGTGATATCTCCACTGACGTAAGGGATGACGCACAATCCCACTATCCT
TCGCAAGACCTTCCTCTATATAAGGAAGTTCATTTCATTTGGAGAGGACACGCTGAAATCACCAGTCTCTCTCTACAAATCTATCTCTCT
CGAGCTTTCGCAGATCCCGGGGGGCAATGAGATATGAAAAAGCCTGAACTCACCGCGACGTCTGTCGAGAAGTTTCTGATCGAAAAGTTC
GACAGCGTCTCCGACCTGATGCAGCTCTCGGAGGGCGAAGAATCTCGTGCTTTCAGCTTCGATGTAGGAGGGCGTGGATATGTCCTGCGG
GTAAATAGCTGCGCCGATGGTTTCTACAAAGATCGTTATGTTTATCGGCACTTTGCATCGGCCGCGCTCCCGATTCCGGAAGTGCTTGAC
ATTGGGGAGTTTAGCGAGAGCCTGACCTATTGCATCTCCCGCCGTGCACAGGGTGTCACGTTGCAAGACCTGCCTGAAACCGAACTGCCC
GCTGTTCTACAACCGGTCGCGGAGGCTATGGATGCGATCGCTGCGGCCGATCTTAGCCAGACGAGCGGGTTCGGCCCATTCGGACCGCAA
GGAATCGGTCAATACACTACATGGCGTGATTTCATATGCGCGATTGCTGATCCCCATGTGTATCACTGGCAAACTGTGATGGACGACACC
GTCAGTGCGTCCGTCGCGCAGGCTCTCGATGAGCTGATGCTTTGGGCCGAGGACTGCCCCGAAGTCCGGCACCTCGTGCACGCGGATTTC
GGCTCCAACAATGTCCTGACGGACAATGGCCGCATAACAGCGGTCATTGACTGGAGCGAGGCGATGTTCGGGGATTCCCAATACGAGGTC
GCCAACATCTTCTTCTGGAGGCCGTGGTTGGCTTGTATGGAGCAGCAGACGCGCTACTTCGAGCGGAGGCATCCGGAGCTTGCAGGATCG
CCACGACTCCGGGCGTATATGCTCCGCATTGGTCTTGACCAACTCTATCAGAGCTTGGTTGACGGCAATTTCGATGATGCAGCTTGGGCG
CAGGGTCGATGCGACGCAATCGTCCGATCCGGAGCCGGGACTGTCGGGCGTACACAAATCGCCCGCAGAAGCGCGGCCGTCTGGACCGAT

GGCTGTGTAGAAGTACTCGCCGATAGTGGAAACCGACGCCCCAGCACTCGTCCGAGGGCAAAGAAATAGAGTAGATGCCGACCGGATCTG
TCGATCGACAAGCTCGAGTTTCTCCATAATAATGTGTGAGTAGTTCCCAGATAAGGGAATTAGGGTTCCTATAGGGTTTCGCTCATGTGT
TGAGCATATAAGAAACCCTTAGTATGTATTTGTATTTGTAAAATACTTCTATCAATAAAATTTCTAATTCCTAAAACCAAAATCCAGTAC
TAAAATCCAGATCCCCCGAATTAATTCGGCGTTAATTCAGTACATTAAAAACGTCCGCAATGTGTTATTAAGTTGTCTAAGCGTCAATTT
GTTTACACCACAATATATCCTGCCA

NbmiR482a target site
AtTAS1c-derived spacer
T-DNA right border
T-DNA left border
*ccd*B gene
*Bsa*I site
Inverted *Bsa*I site
Chloramphenicol resistance gene
attB1
attB2
Nos terminator
CaMV promoter
kanamycin resistance gene
Hygromycin resistance gene
2x35S CaMV promoter
CaMV terminator
